# Solid-State NMR Spectroscopy of Metal–Organic Framework Compounds (MOFs)

**DOI:** 10.3390/ma5122537

**Published:** 2012-11-28

**Authors:** Herbert C. Hoffmann, Marta Debowski, Philipp Müller, Silvia Paasch, Irena Senkovska, Stefan Kaskel, Eike Brunner

**Affiliations:** 1 Department of Chemistry and Food Chemistry, Bioanalytical Chemistry, Dresden University of Technology, Dresden 01062, Germany; E-Mails: herbert.hoffmann@tu-dresden.de (H.C.H.); c9debmar.stud@cclh28.chm.tu-dresden.de (M.D.); silvia.paasch@tu-dresden.de (S.P.); 2 Department of Chemistry and Food Chemistry, Inorganic Chemistry I, Dresden University of Technology, Dresden 01062, Germany; E-Mails: philipp.mueller@chemie.tu-dresden.de (P.M.); irena.senkovska@chemie.tu-dresden.de (I.S.); stefan.kaskel@chemie.tu-dresden.de (S.K.)

**Keywords:** metal–organic frameworks, porous materials, solid-state NMR, host–guest interactions, ^129^Xe NMR

## Abstract

Nuclear Magnetic Resonance (NMR) spectroscopy is a well-established method for the investigation of various types of porous materials. During the past decade, metal–organic frameworks have attracted increasing research interest. Solid-state NMR spectroscopy has rapidly evolved into an important tool for the study of the structure, dynamics and flexibility of these materials, as well as for the characterization of host–guest interactions with adsorbed species such as xenon, carbon dioxide, water, and many others. The present review introduces and highlights recent developments in this rapidly growing field.

## 1. Introduction

Nuclear Magnetic Resonance (NMR) spectroscopy is a well-established tool for the investigation of various types of porous materials. During the past decades, solid-state NMR spectroscopy has been successfully used for characterizing the structure of these materials as well as for studying so-called host–guest interactions with adsorbed species, e.g., xenon, carbon dioxide, and many others including *in situ* studies of catalytic reactions and diffusion processes (see, e.g., [1,2,3,4,5,6,7,8,9,10,11,12,13,14,15,16]).

**Figure 1:**
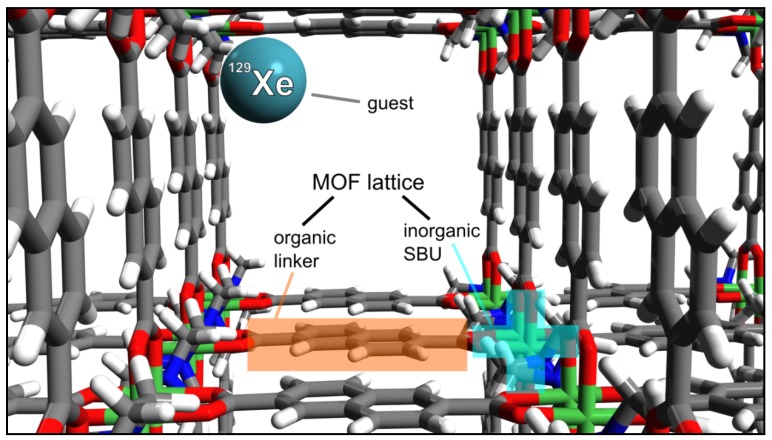
Schematic representation of the metal–organic frameworks (MOF lattice (here: DUT-8(Ni) [23]), consisting of organic linkers and inorganic secondary building units (SBUs). The MOF serves as a host structure for guests such as ^129^Xe within the void spaces. C: grey, H: white, N: blue, O: red, Ni: green, Xe: cyan.

In addition to well-known compounds such as zeolites or activated carbon, an entirely new class of crystalline porous solids with interesting properties such as extraordinarily high specific surface area and gas storage capacity has been developed: metal–organic frameworks (MOFs, see, e.g., [17,18,19,20,21]). These materials are composed of an organic part, so-called linkers, interconnecting the inorganic secondary building units (SBUs) (see Figure 1). Generally, the SBUs include metal ions like Cu, Ni, Zn, or Co which are aggregated into M-O-C clusters by multidentate linker, such as carboxylate. In many MOFs, the metal clusters are discrete, *i.e.*, the inorganic sublattice is zero-dimensional (0D) [21]. That means, the SBUs are not directly connected to each other—but *via* the organic linkers. However, the metal subunit can also be more complex: for example it can consist of inorganic chains leading to a 1D inorganic subnetwork, and subnetworks up to the dimensionality of inorganic subframeworks (3D) are also known [18,21].

The coordinative interactions between inorganic building units and linkers are relatively strong, *i.e.*, of the order of 100 kJ/mol. Therefore, they result in rigid three-dimensional networks, which are often “open”, *i.e.*, they exhibit micro- or mesopores filled with solvent molecules. The solvent can be removed—for example by thermal activation or supercritical drying—while maintaining the network structure of the MOF. In those cases, adsorption of molecules within the resulting pore system is possible. In contrast to the mostly rigid structures of traditional porous materials, some MOFs, e.g., Material Institut Lavoisier-53 (MIL-53, [22]), Dresden University of Technology-8 (DUT-8(Ni), or DUT-8(Co), [23,24]), exhibit a flexible network and show reversible structural transformations during the adsorption/desorption of several gases such as nitrogen, carbon dioxide, or xenon.

**Figure 2:**
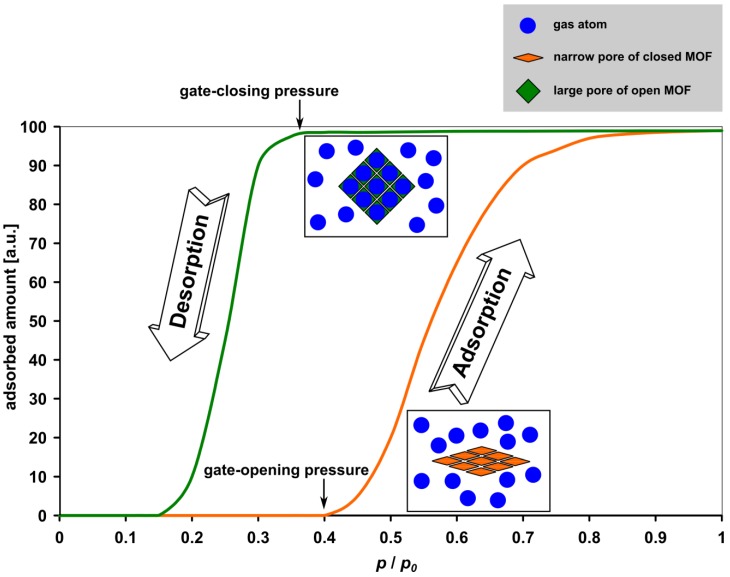
Schematic demonstrating the gate-pressure effect for isothermal adsorption.

This interesting phenomenon is denoted as breathing (MIL-53) or gate-pressure effect (DUT-8(Ni)) and finds increasing interest [25]. After removal of solvent molecules from such flexible gate-pressure compounds, the network transforms to the narrow-pore (or closed) state. In the case of gate-pressure MOFs the pores are entirely inaccessible for the guest molecules at low pressure and the amount of adsorbed gas is almost zero. By higher pressure (so-called gate-opening pressure [26]) the adsorbed amount suddenly rises (see Figure 2). The structural changes of flexible MOFs are associated with a more or less pronounced hysteresis in adsorption/desorption isotherms, depending on the pressure and temperature. Several possible future applications of flexible MOFs can be envisioned in the field of gas storage, gas and solvent purification or catalysis. Due to unique magnetic, optical, and electronic properties, such as nonlinear optical characteristics [27], magnetic spin frustration [28], and magnetic coupling of paramagnetic metal centers [29], MOFs are assumed to be potentially useful for further applications, e.g., for molecular sensing [30] and optical purposes.

In order to study the structure and adsorption behavior of MOFs, single crystal or powder X-ray diffraction (XRD) in combination with adsorption measurements are commonly applied. Structural investigations of MOFs are, however, sometimes challenging. Traditionally, single crystal X-ray crystallographic methods are the preferred method for crystal structure determination. However, the absence of single crystals of sufficient size or quality, internal thermal motions, disorder, and other challenges (see below) limit the applicability of this method. In contrast to XRD, magnetic resonance spectroscopy locally probes the nuclear or electronic spins present in the sample, *i.e.*, the local environment of each single atom. Therefore, magnetic resonance spectroscopy can be considered as complementary to XRD methods. Electron paramagnetic resonance spectroscopy (EPR [31]) and various NMR spectroscopic techniques including diffusion measurements using pulsed field gradient (PFG) NMR [32] as well as ^129^Xe NMR spectroscopy are very useful in combination with XRD. Therefore, increased efforts are made in order to combine powder X-ray diffraction, NMR crystallography [33,34], and molecular modeling [35]; an approach which is referred to as structure elucidation by combining magnetic resonance, computational modeling and diffraction (SMARTER, see, e.g., [36]). Thereby, the chemical shifts can be computed *ab initio* using pseudopotenials as implemented in the gauge-including projector augmented-wave (GIPAW [35]) approach.

Solid-state NMR is able to identify structural parameters and to detect dynamical effects. It is capable of revealing the presence of mobile substructures or molecules. Solid-state NMR experiments for the detection of thermal motions/exchange processes such as one-dimensional exchange spectroscopy by sideband alternation (ODESSA [37,38]) or dipolar centerband-only detection of exchange (CODEX [39,40])—which were previously designed for other materials—may also become useful also for MOFs. Note that the latter experiment is capable of detecting slow motions from milliseconds up to seconds. For the investigation of the framework itself, ^1^H and ^13^C NMR spectroscopy is often used [23,41]. In addition, other nuclei such as ^27^Al [42,43], ^71^Ga [44,45], ^45^Sc [46], and ^67^Zn [47] can be studied by magic-angle spinning (MAS) NMR spectroscopy in order to detect the environment of the central metal atom [48]. These topics will be discussed in Chapter 2.

Solid-state NMR spectroscopy is, furthermore, extremely helpful in characterizing the interactions between the framework and adsorbed species (host–guest interactions including structural changes) which will be the topic of Chapter 3. For example, ^129^Xe NMR spectroscopy of adsorbed xenon [49] is an excellent method for the study of porous materials because a variety of NMR parameters, in particular the ^129^Xe chemical shift, line width, chemical shift anisotropy, and longitudinal relaxation time *T*_1_ are influenced by structural parameters such as pore size, pore shape, composition of the pore walls, and dynamics. The adsorption of other molecules such as carbon dioxide and water can also be studied by NMR spectroscopic techniques.

## 2. Characterization of the MOF Lattice

Within the present chapter, we will discuss NMR spectroscopic methods for the characterization of the MOF structure. Important techniques, such as MAS [50,51], cross polarization (CP [52,53,54,55]), cross polarization with polarization inversion (CPPI [56]), attached proton test (APT [57]), and heteronuclear correlation spectroscopy (HETCOR [58]) will be briefly introduced in the context of metal–organic frameworks. Moreover, it should be noted that numerous well-known liquid-state NMR experiments have meanwhile found solid-state NMR-spectroscopic analogues. Examples are MAS-J-heteronuclear multi-quantum correlation (MAS-J-HMQC [59]), MAS-J-heteronuclear single quantum coherence (MAS-J-HSQC [60]), insensitive nuclei enhanced by polariazation transfer MAS (INEPT MAS [61]), and incredible natural abundance double quantum transfer MAS (INADEQUATE MAS [62]).

**Figure 3:**
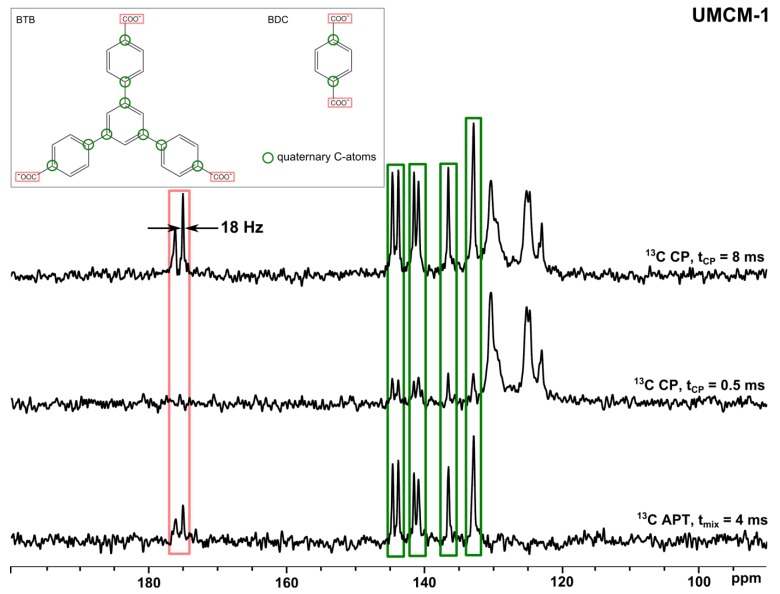
^13^C{^1^H} CP MAS NMR spectra (upper and middle spectrum) and solid-state attached proton test (APT) spectrum (lower spectrum) of activated UMCM-1.

Solid-state NMR spectroscopy offers various possibilities in order to characterize the MOF lattice (see Figure 1) [63,64,65,66,67,68,69]. As already mentioned, the organic parts of the framework can be studied by solid-state ^13^C and ^1^H NMR spectroscopy. Often, the solid-state ^13^C NMR spectra of MOFs exhibit narrow signals, *i.e.*, an excellent spectral resolution. This is demonstrated for the mesoporous MOF University of Michigan crystalline material-1 (UMCM-1) in Figure 3.

To obtain such narrow signals, line broadening has to be effectively suppressed. In non-paramagnetic solids, there are mainly four interactions leading to broadened lines [70,71,72]: The homo- and heteronuclear magnetic dipole-dipole interactions which act directly through space, the chemical shift anisotropy (CSA), and the electric quadrupole interactions for quadrupolar nuclei (*I* > 1/2) [73]. Hence, special techniques have to be used which suppress these line broadening interactions to get well-resolved spectra. The most popular method among these techniques is MAS, *i.e.*, the spinning of the sample around an axis tilted by the magic angle (54.7°) with respect to the external magnetic field, ***B*_0_** [50]. The spectra in Figure 3 are, for example, acquired using a 2.5 mm rotor filled with 3–4 mg of UMCM-1 under sample spinning at 16 kHz. ^1^H decoupling is routinely applied to suppress the influence of the strongly coupled ^1^H nuclei upon the ^13^C signals, typical decoupling sequences are two-pulse phase-modulated decoupling (TPPM) or small phase incremental alteration (SPINAL [74,75]). In the case of half-integer quadrupolar nuclei with *I* > 1/2 such as ^27^Al (*I* = 5/2 [42,43]), ^67^Zn (*I* = 5/2 [47]), ^71^Ga (*I* = 3/2 [44,45]), and ^45^Sc (*I* = 7/2 [46]) MAS does not result in complete removal of the line broadening electric quadrupole interaction due to residual second-order broadening [48,70,73,76]. Typical values for the quadrupolar frequency *C_Q_* and asymmetry parameter *η* observed in MOFs are given in Table 1. This broadening, however, scales with 1/*B*_0_, *i.e.*, the resolution significantly increases in high magnetic fields. Furthermore, special experiments have been designed in order to overcome this problem [76]. The second order quadrupolar line broadening can be suppressed by manipulation of the corresponding spatial or spin components of the second-order quadrupolar Hamiltonian. In the former case, technically sophisticated experiments such as double rotation (DOR [77]) and dynamic angle spinning (DAS [78]) are performed. Multiple quantum MAS (MQMAS [79,80,81]) as well as satellite transition MAS (STMAS [82]) can be applied to influence the corresponding spin terms. The MQMAS experiment results in a two-dimensional spectrum with an isotropic (indirect) spectral dimension and a second spectral dimension containing isotropic contributions as well as the anisotropic line shape (see Equations 12a,b in [79]). The time-domain data can be acquired either in a two- or three-pulse experiment with a corresponding phase cycle [79] in order to select multiple quantum coherence during the evolution period. Note that the data processing requires a so-called shearing transformation apart from the two-dimensional Fourier transform [79]. The MQMAS experiment is meanwhile one of the most frequently applied techniques for the investigation of quadrupolar nuclei (see below). In 2007 Vosegaard and Massiot developed a method called “chemical shift-quadrupolar projection-reconstruction of one-dimensional spectra” (CQ-PRODI [83]). CQ-PRODI is based on the correlation of chemical shifts with the second-order quadrupolar line shapes from multiple spectra recorded at different ***B*_0_** strengths, which allows the construction of high-resolution spectra.

**Table 1:** Examples for the quadrupolar parameters *C_Q_* and *η* as observed for several MOFs.

Nucleus	MOF compound	$\left|C_{Q}\right|$*/*MHz	*η*_Q_	References
^27^Al	MIL-120(Al)	8.1	–	[67]
		4.8	–	[67]
^67^Zn	ZIF-7	6.2	0.95	[47]
	ZIF-8 (as-synthesized)	1.1	0.81	[47]
	ZIF-14	2.8	0.86	[47]
^71^Ga	MIL-96(Ga)	4.8	0.00	[44]
		4.2	0.37	[44]
		3.1	0.52	[44]
	MIL-120(Ga)	14.3	0.17	[45]
		9.4	0.37	[45]
	MIL-124(Ga)	14.1	0.94	[45]

Nuclei such as ^13^C, ^15^N, and ^29^Si with relatively small gyromagnetic ratios and of low natural abundance can be enhanced by cross polarization [52,53,54,55] which is based on the through-space heteronuclear dipole-dipole interaction with neighbouring nuclei, typically ^1^H (see Figure 3). The variation of the CP contact time helps in signal assignment: In the upper spectrum of Figure 3, the CP contact time was 8 ms, which is long enough to transfer polarization to practically all ^13^C atoms which are present. Hence, all signals are detected in this spectrum. In contrast, the middle spectrum has been acquired with a contact time of only 0.5 ms. This time is too short to polarize ^13^C atoms which are relatively distant from ^1^H atoms like quaternary C-atoms. Hence, the corresponding signals are more or less suppressed (see Figure 3, middle spectrum). In the case of the carboxylic groups, no signals are observable at all due to the large distance between these ^13^C-atoms and ^1^H nuclei. Another helpful tool to distinguish between different C-species is the acquisition of either CPPI [56] or solid-state APT [57] spectra (see Figure 3, lower spectrum). In APT spectra, the signals of quaternary C-atoms and CH_2_-groups are positive and the signals of CH-/CH_3_-groups are negative. However, the signals of quaternary C-atoms usually exhibit the highest intensities whereas the other signals are often weak or fully suppressed, as in Figure 3 (lower spectrum)

**Figure 4:**
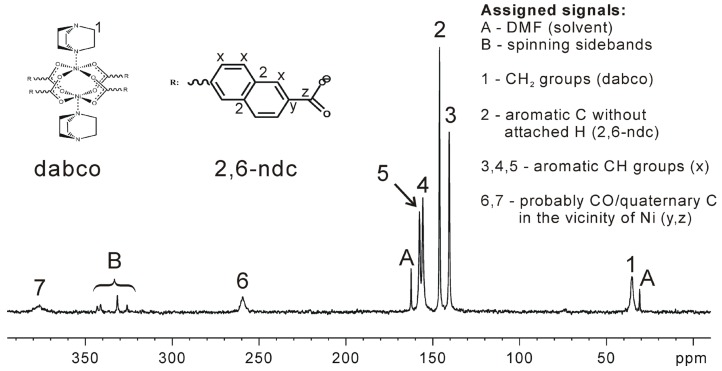
Assignment of the signals to the carbon atoms present in resolvated DUT-8(Ni) (Reproduced with permission from [24], copyright 2012 the Royal Society of Chemistry).

Note, that the metal centers—especially in case of paramagnetic species such as Ni, Co, or Cu—may strongly influence chemical shifts of neighboring nuclei such as ^1^H or ^13^C. DUT-8(Ni) exhibits a gate pressure effect, which is accompanied by a color change and changes in the magnetization. Figure 4 [24] shows that the Ni sites obviously result in unusually high chemical shifts of neighboring atoms.

**Figure 5:**
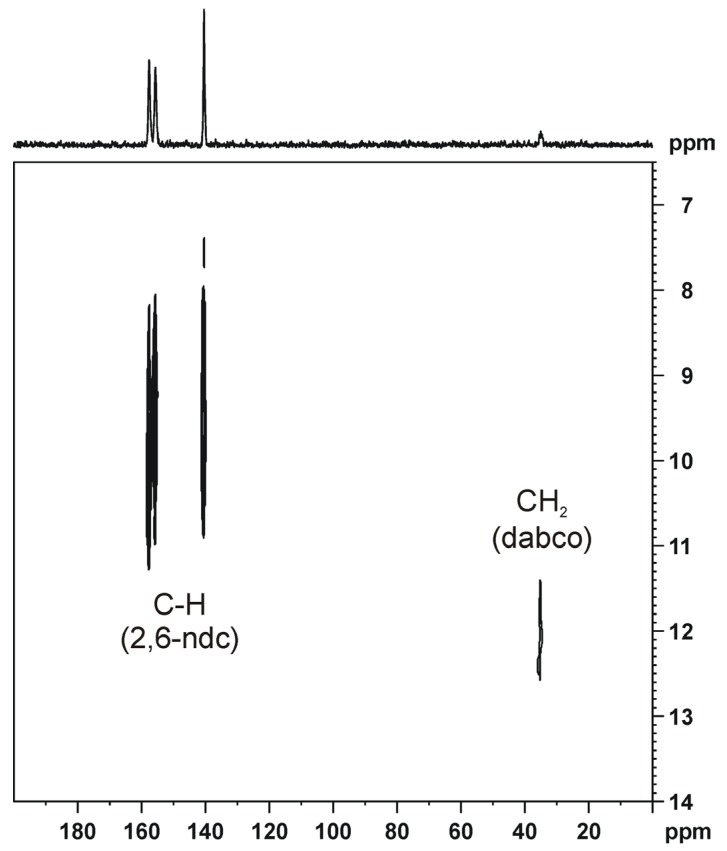
^13^C{^1^H} HETCOR spectrum of resolvated DUT-8(Ni) (Reproduced with permission from [24], copyright 2012 the Royal Society of Chemistry). The spectrum was measured using FSLG decoupling during the ^1^H evolution period t_1_.

If the NMR signals of the considered sample are intense enough—as in the case of DUT-8(Ni)—HETCOR spectra [58,84,85] can be measured. In such experiments, ^1^H and ^13^C atoms are correlated by polarization transfer, e.g., via the heteronuclear dipolar coupling. The HETCOR spectrum of DUT-8(Ni) is shown in Figure 5 [24]. Compared to the dissolved pure linker (dabco), the corresponding ^13^C signal is shifted by *ca.* 10 ppm to lower chemical shifts. In contrast, the ^1^H signal of the CH_2_ group of dabco is shifted by *ca.* 10 ppm to higher chemical shifts. These effects are likely due to the influence of the close Ni sites. Note, that the mixing time for the HETCOR spectrum shown in Figure 5 was only 200 µs. Therefore, the quaternary C-atoms of the linker (2,6-ndc, see Figure 4 [24]) are not observable in this spectrum. It should be noted that heteronuclear ^1^H-X correlation spectra (X = ^13^C, ^15^N, ^29^Si, ^31^P, ...) are often acquired under homonuclear decoupling during the ^1^H evolution period. The reason is the relatively large residual line width of ^1^H MAS NMR signals which is dominated by the homonuclear magnetic dipole-dipole interaction. The resulting line width contribution can be written as [86,87]:

(1) $$\Delta \nu_{1/2}^{\text{MAS}}=\frac{1}{A}\frac{{\left(\Delta \nu_{\text{II}}\right)}^{2}}{\nu_{\text{r}}}$$

Δν_II_ is the homonuclear contribution to the static line width, *i.e.*, the line width without MAS. The geometry-dependent factor *A* typically amounts to *ca.* 10–40 [86,87]. Complete suppression of the homonuclear magnetic dipole-dipole interaction in strongly coupled spin systems would require very fast sample spinning well beyond 100 kHz [86,88] which is not yet accessible. This is the reason why directly detected ^1^H MAS NMR spectra of such systems are often poorly resolved. Especially in multidimensional NMR experiments, frequency-switched or phase-modulated Lee-Goldburg decoupling (FSLG/PMLG [89,90,91,92]) or decoupling using mind-boggling optimisation (DUMBO [93,94]) are, therefore, usually applied to suppress the homonuclear magnetic dipole-dipole interaction.

**Figure 6:**
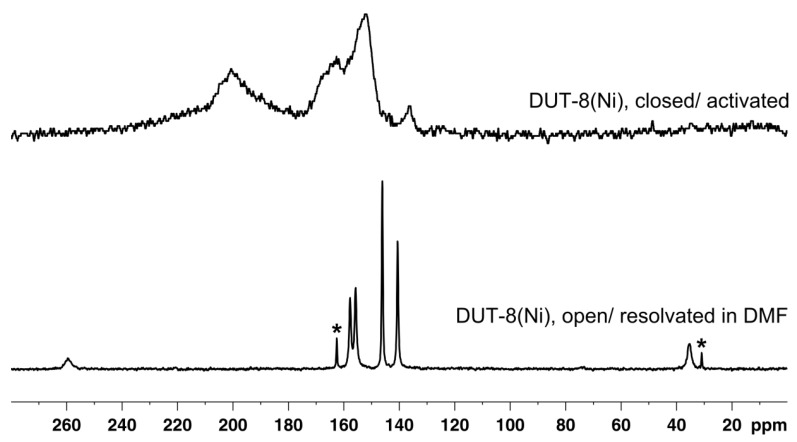
^13^C{^1^H} CP MAS NMR spectra of DUT-8(Ni) in different states. In the closed state (upper spectrum), the lines are broadened and shifted, most likely due to the increasing paramagnetism of the Ni sites. Signals due to DMF are denoted by asterisks.

The structural transition of DUT-8(Ni) to the narrow-pore state results in pronounced signal shifts and line broadening in the ^13^C{^1^H} CP MAS NMR spectrum (see Figure 6).

**Figure 7:**
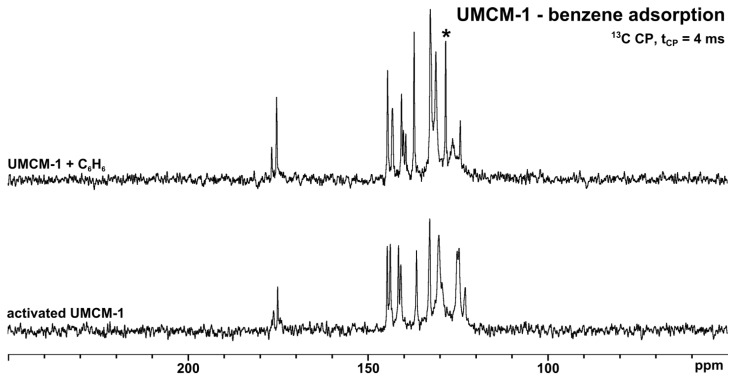
^13^C{^1^H} CP MAS NMR spectra of activated and C_6_H_6_-loaded UMCM-1, respectively. The signal due to C_6_H_6_ is denoted by an asterisk.

Adsorbed molecules can also influence several NMR parameters of nuclei located on the MOF lattice, especially the chemical shifts, line widths, and longitudinal relaxation times. Furthermore, if the adsorbed molecules are sufficiently immobilized, *i.e.*, if their interactions with the MOF lattice are strong enough, the signals of the adsorbed molecules are also observed in CP spectra. For example, the signal at 128 ppm in the upper spectrum of Figure 7 is due to benzene (C_6_H_6_).

Solid-state NMR spectroscopy can also be applied to test the incorporation of the desired linkers or certain structural groups into the MOF lattice [95]. NMR spectroscopy was, furthermore, used to prove the space group derived from X-ray data [96]. The idea behind this is that the number of signals from one single atom is equal to the number of crystallographically non-equivalent positions for the given atom.

Shi *et al.* applied ^31^P and ^23^Na MAS NMR spectroscopy in combination with XRD measurements [97]. ^23^Na MAS NMR was used to quantify the amount of sodium ions which are present in the MOF channels, because the distinction between residual H_2_O molecules and sodium ions inside the MOF channels was complicated in standard XRD experiments. Two crystallographically non-equivalent Na^+^ sites with different occupation numbers have been observed. ^23^Na triple-quantum (3Q) MAS spectra [98] were acquired using the z-filtered three-pulse sequence (two hard pulses and one final soft pulse) [98]. The so-called soft-pulse-adding-mixing (SPAM [99]) through multiple coherence selection served for sensitivity enhancement [98]. The spectra delivered further structural information about the sodium ions as a function of the hydration degree. In the hydrated state, the sodium ions are disordered. In contrast, only one single state is observed in the dehydrated state. Furthermore, the quadrupolar coupling constant of ^23^Na significantly depends on the hydration state which is explained by the influence of the water content upon the mobility of Na^+^.

NMR spectroscopy is also able to help understanding the formation mechanisms of MOFs as demonstrated by Bajpe *et al.* [100]. The authors used liquid-state ^17^O, ^31^P and ^183^W NMR spectroscopic methods to deeply investigate the template-driven synthesis of Cu_3_(BTC)_2_ (Hong Kong University of Science and Technology-1, HKUST-1) using Keggin-type heteropolyacids as templates on the molecular scale [100].

Solid-state NMR spectroscopy is, furthermore, capable of delivering spatial information. Heteronuclear magnetic dipole-dipole interactions can be measured selectively by the so-called rotational echo double resonance (REDOR [101]) experiment. Re-introduction of homonuclear interactions can be obtained in rotational resonance experiments [102]. Meanwhile, various dipolar recoupling schemes have been designed in order to selectively exploit homonuclear as well as heteronuclear magnetic dipole-dipole interactions (see, for example [103,104,105,106] and references therein). Such techniques can, e.g., be used to determine the spatial proximity and orientation of the linkers relative to each other as well as with respect to adsorbed species (see below). Methods such as ^1^H-^1^H homonuclear dipolar recoupling back-to-back (BABA) and ^1^H–^1^H radio frequency dipolar recoupling (RFDR) were shown to be well suited for such purposes [63,107,108].

**Figure 8:**
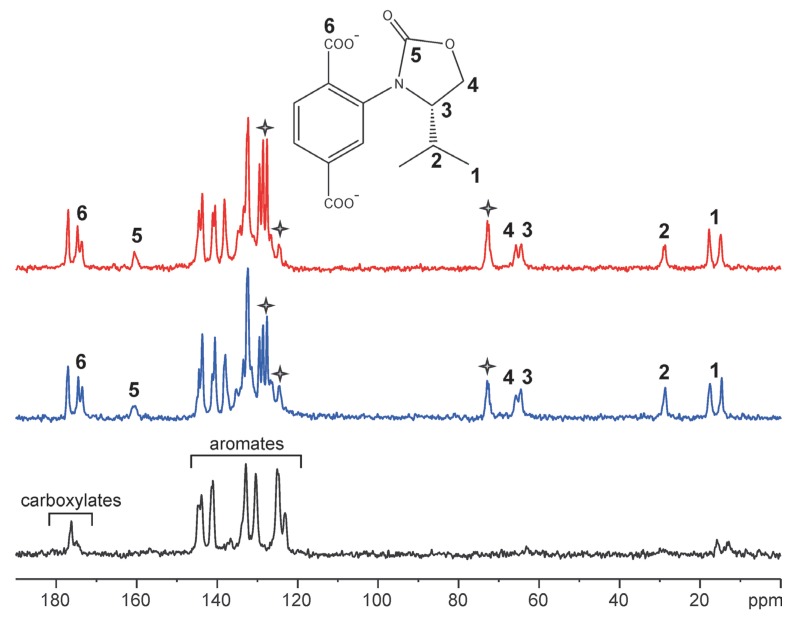
^13^C CP MAS NMR spectra of *i*Pr-ChirUMCM-1. Top (red): Sample loaded with (*R*)-TFPE. Middle (blue): Sample loaded with (*S*)-TFPE. Bottom (black): Unloaded sample. The structure of the modified BDC-linker (Chir-BDC) bearing the chiral side group is shown in the figure in order to define the carbon positions of the assigned signals. Stars denote signals due to TFPE (Reproduced with permission from [138], copyright 2012 the Royal Society of Chemistry.)

Post- or presynthetic functionalization of MOFs is a very promising area of MOF research [109,110,111,112,113,114,115,116,117,118,119,120,121,122,123,124,125,126]. Ahnfeldt *et al.* [125] made use of solid-state heteronuclear ^1^H–^15^N, ^1^H–^13^C, and homonuclear ^1^H–^1^H correlation spectroscopy in order to prove the methylation of NH_2_ groups in Christian-Albrechts-University-1 (CAU-1) derivatives. Pt nanoparticles were introduced in MOF-177 by loading with a volatile Pt precursor and subsequent reduction [126]. The success of the loading process, *i.e.*, the incorporation of the organic Pt precursor as well as the integrity of the host lattice after the treatment could be monitored by ^13^C MAS NMR spectroscopy. Often, the functional groups attached to the linkers are disordered and/or exhibit a highly dynamical behaviour. Due to this effect, the functional groups—which are of special interest—cannot be localized in the structure by standard XRD techniques in such cases. To prove the functionalization by NMR spectroscopy, two different ways are described in the literature. The MOF can be dissolved and the functional groups become then observable *ex situ* by liquid-state NMR spectroscopy as described in [127,128,129,130,131]. On the other hand, the functional groups can be detected *in situ* by MAS NMR spectroscopy [42,132,133]. The advantage of the *in situ* method is its non-destructive character. Furthermore, information about the mobility of the functional groups is available from solid-state NMR measurements. In cases where the linkers are not fully functionalized and/or the functional groups are distributed statistically over the MOF lattice [134,135] like in MIXMOFs [136], NMR spectroscopic methods can deliver information about the degree of functionalization which helps to evaluate the efficiency of the applied postsynthetic functionalization techniques [42,109,128,130]. Recently, chiral UMCM-1 was synthesized by the pre-synthetic attachment of chiral auxiliaries to the linkers [137]. These chiral side groups are rather mobile and disordered in the unloaded MOF. However, the corresponding ^13^C NMR signals become clearly visible after loading the samples with a chiral shift agent (1-phenyl-2,2,2-trifluoroethanol, TFPE) due to the interaction between the side groups and TFPE molecules ([138], see Figure 8). Moreover, chiral discrimination becomes possible in analogy to the well-known liquid-state NMR spectroscopic methodology [139] because the chemical shift of several ^13^C nuclei located at the chiral side groups was found to depend on the enantiomeric form of the linkers and the shift agent by forming NMR-spectroscopically distinguishable diastereomeric complexes [138].

In addition to the characterization of the organic moieties in MOFs, solid-state NMR spectroscopy can also help to investigate the inorganic SBUs, *i.e.*, metal ions or clusters. It is, for example, possible to distinguish between different structural variants of the metal centres [140]. Ravon *et al.* generated statistically distributed vacancies at the SBUs, *i.e.*, they created SBUs without an organic linker at one or more coordination sites [141]. The goal of this procedure was the introduction of Brønsted acid sites for catalytic purposes—an approach which is generally used to form MOFs with coordinatively unsaturated sites (cus) [142,143]. NMR spectroscopy was then applied in order to prove the formation of such vacancies and to quantify their amount as a function of the synthesis parameters.

Solid-state NMR spectroscopy also serves to prove structural models of the SBU sublattice. The coordination environment can be detected using chemical shifts and/or quadrupolar coupling constants for metal atoms such as ^27^Al [107,108,144], ^71^Ga [44], ^45^Sc [46,132], and ^6^Li [145]. Furthermore, metal atoms outside the MOF lattice, *i.e.*, located in the pores can be probed by MAS NMR. Other authors tried to insert lithium ions into the pores of anionic MOFs by means of cation exchange. ^7^Li NMR can then contribute to an understanding of the coordination state of the lithium ions, for example, as a function of the degree of solvation [146].

NMR spectroscopy can also be applied to study properties such as the proton conductivity [147,148,149] or other dynamical phenomena. The organic linkers in MOFs exhibit a relatively high mobility [150,151]. This internal or intrinsic dynamic has been studied using ^2^H NMR spectroscopy by several groups [132,152,153,154,155]. Such experiments enable the investigation of molecular dynamics proceeding at the microsecond timescale [132,153].

Finally, it should be noted that recent methodological developments, such as the improvement of hyperpolarization techniques, in particular dynamic nuclear polarization (DNP [156,157,158,159,160,161,162,163]) are likely to open completely new possibilities for solid-state NMR experiments on metal–organic frameworks. Dynamic nuclear polarization relies on the transfer of polarization from an electron spin to nuclear spins [156]. In the case of diamagnetic samples, doping with appropriate paramagnetic compounds is necessary [159]. Due to the inherently much higher magnetic moment of electrons, their spin polarization is correspondingly high and can, therefore, be used to enhance the nuclear spin polarization. This results in a significant sensitivity enhancement of the corresponding NMR spectra and is, meanwhile, feasible in high magnetic fields and under magic angle spinning conditions [157,160,161,162,163]. Recently, DNP has also been used to enhance the solid-state NMR signals in the MOF MIL-68 [163]. The sample was impregnated with an EtCl_4_ solution containing 16 mM of the exogenous biradical bTbK [159]. Signal enhancements of approximately one order of magnitude could be observed for the ^13^C{^1^H} CP MAS NMR spectra. This significant improvement corresponds to two orders of magnitude shorter acquisition times and is of special importance especially with respect to multidimensional experiments.

## 3. Solid-State NMR Spectroscopic Studies of Host–Guest Interactions

Apart from the structural characterization of the MOF lattice itself, adsorption processes and adsorption-induced structural changes, *i.e.*, host–guest interactions are of special importance. Solid-state NMR spectroscopy is particularly well suited to study interactions between porous materials and adsorbed atoms/molecules. This is due to a number of advantageous properties:

(1) NMR does not require any kind of long-range order;
(2) NMR is capable of providing local structural information including distance measurements;
(3) NMR allows determining the flexibility as well as adsorption-induced structural changes of the host;
(4) NMR is capable of determining the mobility of the host lattice, *i.e.*, the dynamics as well as the mobility of the adsorbed species.

The resulting possibilities of solid-state NMR spectroscopy with respect to host–guest interactions in MOFs will be highlighted and demonstrated in the present chapter on a number of selected examples.

### 3.1. Host–Guest Interactions in MOFs Studied by ^129^Xe NMR Spectroscopy

^129^Xe has been introduced as a probe into surface NMR spectroscopy by Ito and Fraissard [49,164]. Its use for surface NMR studies was further facilitated by the introduction of hyperpolarization techniques, especially spin-exchange optical pumping (SEOP [165,166,167,168]). Meanwhile, xenon has found numerous applications for the characterization of various types of porous materials (see, e.g., [169,170,171,172,173,174,175,176,177,178]). ^129^Xe NMR spectroscopy provides a variety of interesting parameters encoding information about the surface and pore systems under study. Such parameters are the chemical shift, the line width, the chemical shift anisotropy, and the longitudinal relaxation time *T*_1_. A first application of ^129^Xe NMR spectroscopy to MOFs has been reported in 2006 [179]. Subsequently, other authors made use of the favorable properties of ^129^Xe NMR in order to study MOFs [23,180,181,182,183].

Various parameters influence the ^129^Xe chemical shift of adsorbed xenon gas which can be written as follows [49,164]:

(2) $$\delta =\delta_{0}+\delta_{\text{S}}+\delta_{\text{Xe}-\text{Xe}}+\delta_{\text{M}}$$

*δ*_0_: chemical shift of xenon gas (reference). *δ*_S_: chemical shift due to interaction between xenon atoms and the walls of pores and cages. *δ*_M_: shift contribution caused by paramagnetic sites.

*δ*_Xe–Xe_ = Δ_Xe–Xe_∙ρ_Xe_ is the chemical shift contribution due to Xe–Xe interactions. ρ_Xe_ denotes the density of xenon atoms and Δ_Xe–Xe_ is the slope of *δ*_Xe–Xe_. The possible influence of strongly adsorbing sites as well as of the electric field of cations is considered to be part of *δ*_S_ within the present paper. It was found for zeolites that *δ*_S_ increases if the void space in the pores, *i.e.*, the pore size decreases [184]. Similar correlations can also be observed for silica-based materials in general [178].

**Figure 9:**
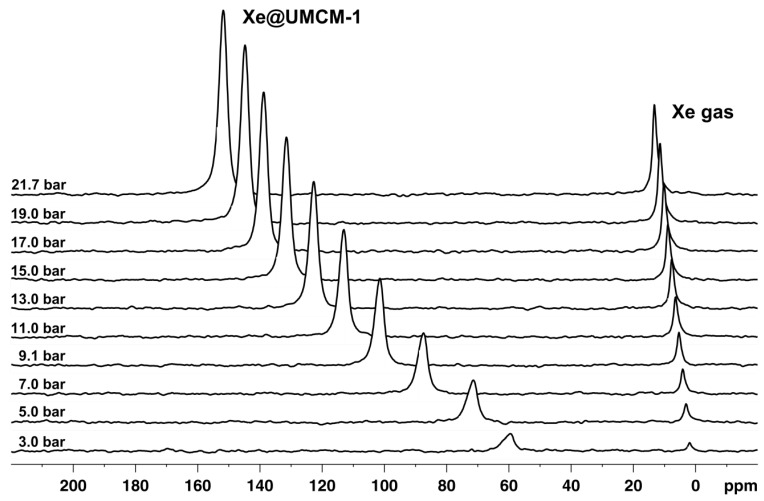
^129^Xe NMR spectra of xenon adsorbed on UMCM-1 measured at room temperature (RT) for various pressures.

For illustration of the major effects observed by ^129^Xe NMR on MOFs, the ^129^Xe NMR spectra of UMCM-1 [185] measured at room temperature as a function of pressure are shown in Figure 9. The spectra are referenced to the chemical shift of gaseous xenon extrapolated to zero pressure, which is set to *δ*_0_ = 0, the commonly used convention. The chemical shift of the gas phase signal depends almost linearly on the pressure in the considered pressure range up to *ca.* 20 bar. Since the xenon pressure is proportional to the density in good approximation for this pressure and temperature range, this observation is in agreement with previous publications predicting a linear interdependence between the chemical shift and the xenon density up to *ca.* 100 amagat [186,187]. One amagat is defined as the number density of gas atoms/molecules per unit volume of an ideal gas at 101.325 kPa and a temperature of 273.15 K.

**Figure 10:**
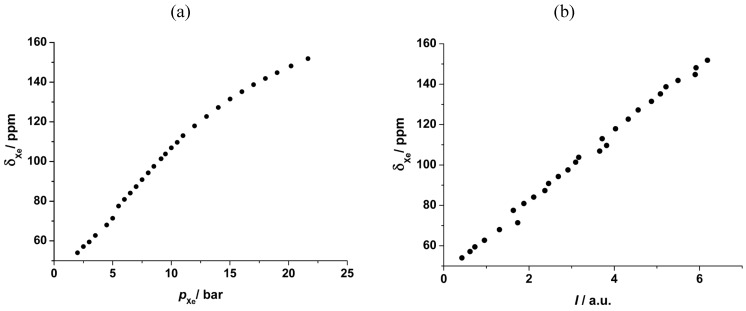
^129^Xe NMR chemical shift of xenon adsorbed on UMCM-1 (see Figure 9) measured at RT as a function of pressure (**a**) and xenon signal intensity (**b**).

**Figure 11:**
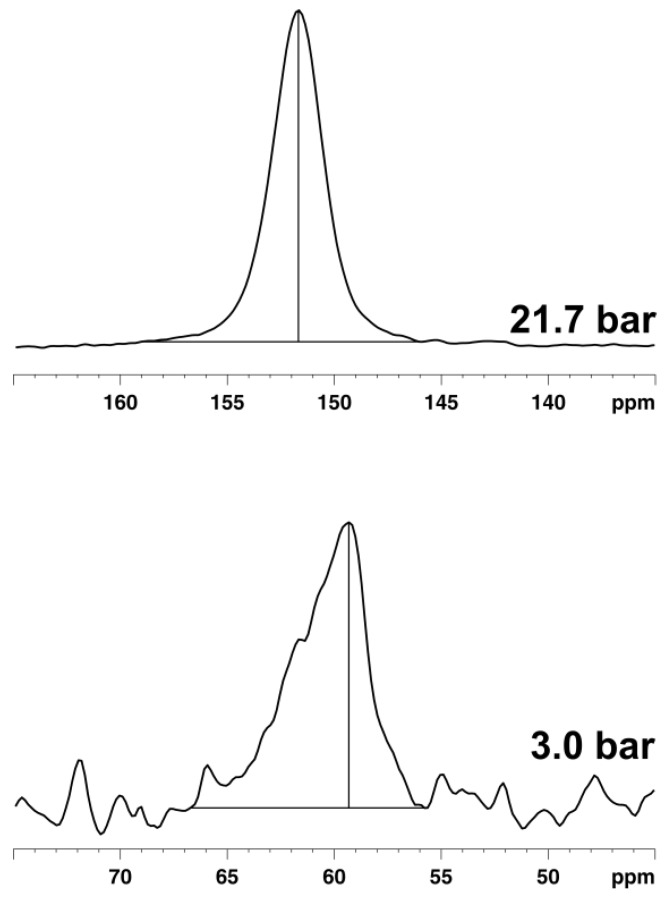
^129^Xe NMR signals of Xe@UMCM-1 measured at RT and different pressures.

**Figure 12:**
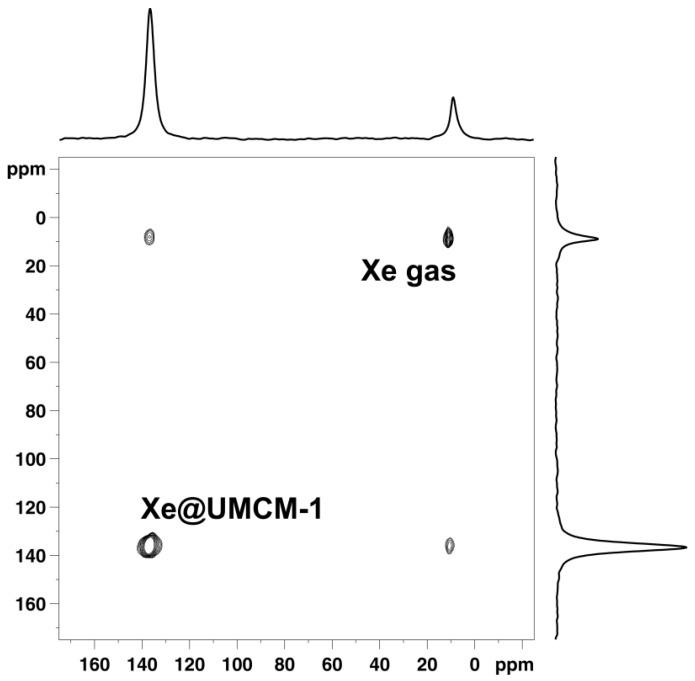
2D ^129^Xe EXSY spectrum of Xe adsorbed on UMCM-1 measured at RT and 70 ms mixing time.

Furthermore, the signal due to xenon adsorbed in the pore system also grows and shifts because the xenon density ρ_Xe_ inside the pore system increases with the xenon pressure (see Figure 9). In the case of UMCM-1, an almost linear interdependence between the chemical shift of adsorbed xenon and the applied pressure is observed up to *ca.* 12 bar at RT (see Figure 10a). Beyond, the slope of the curve decreases significantly. Since the signal intensity is a measure for the xenon concentration inside the (rigid) pore system of UMCM-1, the ^129^Xe NMR chemical shift of the adsorbed xenon should be linearly correlated with the signal intensity according to Equation (2). This is indeed observed (see Figure 10b) which confirms the validity of the equation *δ*_Xe–Xe_ = Δ_Xe–Xe_∙ρ_Xe_ under the present experimental conditions. Extrapolation to zero signal intensity, *i.e.*, zero xenon density yields an extrapolated value of *δ*(0) = 49 ± 3 ppm. If paramagnetic sites were present in UMCM-1, the paramagnetic shift *δ*_M_ would contribute to the latter value. Since UMCM-1 does not contain paramagnetic sites, *δ*(0) represents the xenon-lattice interaction contribution *δ*_S_ (see Equation (2)). It is, furthermore, interesting to note that UMCM-1 exhibits two different types of cavity which are both large enough to host xenon and should be accessible. However, only one signal due to adsorbed xenon is observed. Obviously, the xenon atoms can rapidly exchange between these two cavities. At higher xenon pressures, the signal is narrow and symmetric. However, the signal exhibits an increasingly pronounced anisotropy below *ca.* 5 bar xenon pressure (see Figure 11). This phenomenon is in general observed for xenon in anisotropic local environments and was first described by Springuel-Huet and Fraissard [188]. Much more pronounced anisotropies could be observed previously for the metal–organic framework MIL-53(Al) [182] and other materials with narrow-pore channels (see, e.g., [172,174]). Moreover, the exchange of xenon between the adsorbed state inside the UMCM-1 pore system and the gas phase surrounding the crystallites can be visualized by 2D exchange spectroscopy (EXSY, see Figure 12).

It should be noted that the effects demonstrated above for UMCM-1 are characteristic for porous materials with ***rigid pore systems***. However, metal–organic frameworks can exhibit rather ***flexible pore systems*** (see, e.g., [189,190]). One way of studying the breathing transitions is by sensing the MOF lattice itself and its changes. Since the breathing transitions change the pores and/or their “entries” if the crystal structure of the MOF changes, these effects can also be explored in an indirect way by using probes like ^129^Xe sensing the inner surface of the pores as has been demonstrated for the metal–organic frameworks MIL-53 [182] and DUT-8 [24,183].

MIL-53 is capable of switching between a large-pore and a narrow-pore state depending on the external conditions. It exhibits an interesting temperature-pressure phase diagram [190]. At xenon partial pressures below 1.5 bar, MIL-53 adopts its large-pore state beyond room temperature and switches into the narrow-pore state at decreasing temperatures. Below a loading-dependent threshold temperature, however, it switches back into the large-pore state. In contrast, the large-pore state is always present at elevated pressures beyond *ca.* 1.5 bar. These transitions could be visualized by ^129^Xe NMR spectroscopy [182] because they are accompanied by corresponding changes of the isotropic chemical shift and chemical shift anisotropy of the ^129^Xe NMR signals. The narrow-pore state exhibits a considerably larger isotropic chemical shift and chemical shift anisotropy for ^129^Xe than the large-pore state.

**Figure 13:**
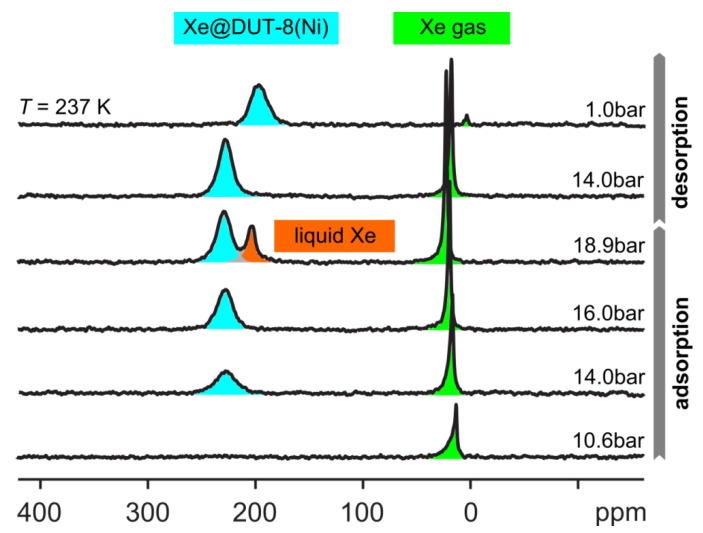
^129^Xe NMR spectra of DUT-8(Ni) pressurized with various amounts of xenon, measured at 237 K. Note that the initially closed structure opens during the adsorption experiment at a gate-opening pressure of *ca.* 12 bar whereas it remains open during desorption down to 1 bar (hysteresis). Reprinted with permission from [183]. Copyright 2011 American Chemical Society.

DUT-8(Ni) is a MOF with a very pronounced gate-pressure effect. Below the temperature-dependent gate-opening pressure, DUT-8(Ni) was found to be entirely “closed” for xenon [183] provided the solvent was previously removed by solvent exchange with subsequent drying using supercritical CO_2_ [191]. Otherwise, the samples are in a narrow-pore state below the gate-opening pressure [23]. Supercritical CO_2_ extracted samples, however, do absolutely not allow xenon penetration into the material below the gate-opening pressure. At the gate-opening pressure, the framework suddenly opens and takes up high amounts of xenon as could be detected by high-pressure *in situ* ^129^Xe NMR spectroscopy (see Figure 13, [183]). Below the gate-opening pressure, only the gas phase signal is observed. Beyond the gate-opening pressure of *ca.* 12 bar at 237 K, a signal at *ca.* 227 ppm occurs. This chemical shift remains almost constant if the pressure is further increased. It amounts to 229 ppm at the highest applicable pressure. This shows that the xenon density ρ_Xe_ inside the pore system remains practically constant during the pore opening process. The chemical shift of adsorbed xenon even exceeds that of liquid xenon (203 ppm at 237 K, see Figure 13). This is indicative for a high xenon density ρ_Xe_. Moreover, a paramagnetic contribution may further enhance the chemical shift. Molecular dynamics simulations were capable of reproducing these structural transitions only if van der Waals interactions were included explicitly into the calculations [183] in agreement with previous theoretical studies on MIL-53 [192,193,194,195]. This observation emphasizes the importance of van der Waals interactions for the characteristic adsorption-induced breathing transitions observed in numerous MOF compounds. It should be noted that the exchange of Ni by Cu, Zn, or Co completely changes the switching behavior of DUT-8 [24]. This observation illustrates that the type of metal center is of crucial importance for the breathing transitions.

### 3.2. Adsorption of Other Guest Molecules on MOFs

The adsorption of gases such as CO_2_ and H_2_ on porous materials is of special importance. Gas separation, e.g., the separation of CO_2_ from industrial exhaust gases is a potentially important field of application for MOFs. Space-, weight-, and cost-efficient H_2_ storage is a yet unsolved problem with respect to the use of H_2_ as a fuel. MOFs are promising candidates for gas storage due to their huge internal surface area which strongly exceeds even the large internal surface of zeolites.

The adsorption of CO_2_ and other molecules on metal–organic framework compounds was studied theoretically, especially with respect to breathing effects on MIL-53 [193,194,195]. In analogy to the behaviour reported above for xenon, the adsorption/desorption of CO_2_ also causes structural transitions in MIL-53.

First ^13^C MAS NMR spectroscopic studies of CO_2_ adsorbed on zeolites were reported in 1974 by Stejskal *et al.* [196]. The isotropic ^13^C chemical shift of physisorbed ^13^CO_2_ is not very sensitive—in contrast to the very sensitive isotropic ^129^Xe chemical shift of xenon (Section 3.1). The signals of physisorbed CO_2_ in micropores are usually shifted by only a few ppm with respect to the gas phase signal which is usually observed at *ca.* 126 ppm (see, e.g., [197,198]). However, the pronounced anisotropy of the CO_2_ molecule results in a correspondingly high ^13^C NMR chemical shift anisotropy Δ = *δ*_⊥_ − *δ*_||_ of *ca.* 335 ppm [199,200]. In the case of spatially anisotropic or restricted motions of CO_2_ in the adsorbed state, the ^13^C NMR signals exhibit characteristic line shapes allowing conclusions about the motional state of the adsorbed molecules [197,198,201]. For example, the rotation axis of the CO_2_ molecules in the molecular frame can be determined [197,201]. Kong *et al.* [201] studied the interaction between CO_2_ and open Mg sites in Mg-MOF-74 giving rise to exceptional CO_2_ capture properties. Using enriched ^13^CO_2_ at low loadings (0.3–0.5 CO_2_/Mg), it was observed that the CO_2_ molecules perform uniaxial rotation around an axis tilted with respect to the symmetry axis of CO_2_. Line shape analysis of the ^13^C NMR spectra allowed measuring this tilt angle. Values in the interval between 56° and 69° were measured depending on the temperature.

**Figure 14:**
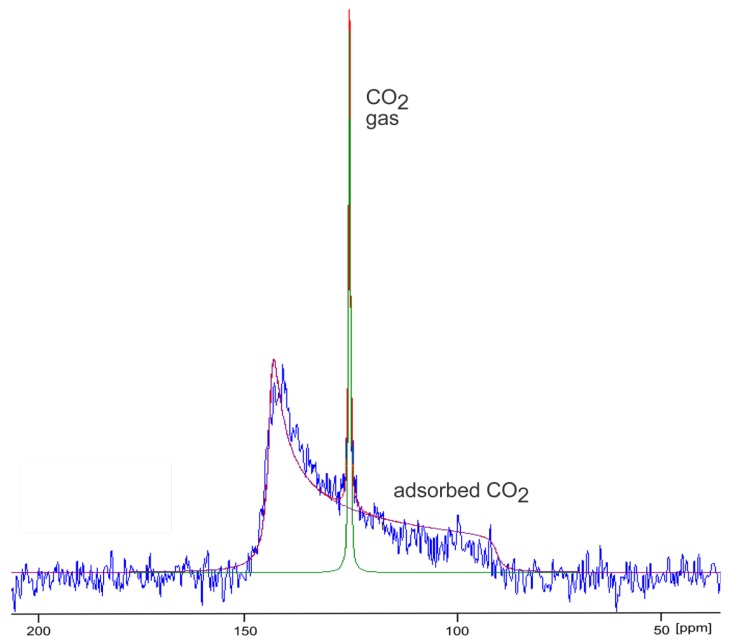
^13^C NMR spectrum of DUT-8(Ni) pressurized with 9.5 bar carbon dioxide measured at 237 K. Note that the initially closed structure opens during the adsorption experiment at temperature-dependent gate-opening pressure (*ca.* 5 bar at 237 K) whereas it remains open during desorption down to 1 bar (hysteresis). (Blue: experimental spectrum, green: simulated gas phase signal, magenta: simulated signal of adsorbed CO_2_; red: Sum of the simulated signals.) The Figure has been prepared using DMFit [202].

These characteristic line shapes are also found in the high-pressure *in situ* ^13^C NMR spectra of CO_2_ adsorbed on the ***flexible*** gate-pressure MOF DUT-8(Ni) (see Figure 14). 

Below the gate-opening pressure, only a signal at 126 ppm occurs which is due to gaseous CO_2_ in the inter-particle space. Beyond the gate-opening pressure, *i.e.*, in the open state of the framework, a broad signal with the characteristic shape caused by chemical shift anisotropy occurs apart from the aforementioned signal due to gaseous CO_2_. Line shape analysis of the former signal reveals a chemical shift anisotropy Δ_av_ of 52 ppm. The significant deviation from the value of 335 ppm for immobilized CO_2_ is caused by motional narrowing. For axially symmetric molecules with axially symmetric chemical shift tensors, the tilt angle θ between the symmetry axis and the rotation axis of the molecule can be estimated using the equation:

(3) $$\Delta_{\text{av}}=\Delta \left(\frac{3\cos^{2}\theta -1}{2}\right)$$

This equation predicts a tilt angle of 49° ± 2° for the molecules adsorbed in the flexible MOF DUT-8(Ni). That means, a certain degree of order is observed for all the CO_2_ molecules adsorbed inside DUT-8(Ni). The tendency of the framework to close the pores must be overcome by the adsorbed molecules as already described for xenon [183]. In the case of the non-spherical CO_2_ molecules, these interactions seemingly result in a certain degree of order for the adsorbed molecules inside the pores.

The interaction between CO_2_ and CD-MOF-2 was studied recently by Gassensmith *et al.* [203] ^13^C MAS NMR spectroscopy was capable of detecting reversible CO_2_-chemisorption in this MOF as could be monitored by the appearance of corresponding signals at 158 ppm.

Molecular hydrogen (H_2_) occurs in two co-existing forms, ortho-hydrogen (o-H_2_, nuclear spin *I* = 1) and para-hydrogen (p-H_2_, *I* = 0). That means, ^1^H NMR exclusively detects o-H_2_. The ortho/para ratio depends on the temperature and pressure. The relative amount of the NMR-active o-H_2_ is *ca.* 75% under standard conditions and decreases with decreasing temperature. Grzech *et al.* [204] have recently studied the adsorption of H_2_ on the metal–organic framework compound Cu_3_(BTC)_2_ and observed the irreversible chemisorption of 1.1 wt % H_2_ at temperatures between 323 K and 423 K at 2 bar H_2_ pressure. ^1^H NMR in combination with IR spectroscopy revealed that the BTC linkers became hydrogenated and were thus converted into their acidic form.

Diffusion of hydrocarbons in MOF-5 was studied by pulsed field gradient (PFG) NMR spectroscopy [32], a well-established technique for the measurement of intracrystalline self-diffusion in porous materials [12]. These studies suggest a superior, very fast diffusion of the studied hydrocarbons within the pores of MOF-5 as well as rapid exchange with the external gas. The latter observation agrees with the observed exchange of xenon between the adsorbed state and the surrounding gas at the time scale of tens of milliseconds (Section 3.1, Figure 12).

NMR spectroscopy is very sensitive with respect to subtle structural changes [140,205]. Thus, it can be used to investigate the reversibility of processes [97] and also to check decomposition processes and phase transitions [206]. Esken *et al.* investigated the very popular MOF-5 by means of ^1^H and ^13^C MAS NMR spectroscopy [207] after loading with metal–organic precursors and subsequent H_2_ treatment. The structural integrity after this treatment could be proven. Similar methods were successfully applied to other systems as well [208]. Especially in cases where amorphous phases are generated, NMR spectroscopy turned out to be very helpful [204,209,210].

Volkringer *et al.* characterized MIL-121 in both the as-synthesized and the activated form [63]. Information about spatial proximities and interactions of the linkers and guest molecules could be obtained from ^1^H-^1^H single quantum-double quantum (SQ-DQ), BABA, ^1^H-^1^H SQ-SQ RFDR, ^27^Al{^1^H} HETCOR, and ^27^Al MQMAS methods. The authors observed the formation of water clusters in the pores of activated MIL-121 during spontaneous rehydration. Interestingly, these clusters seem to hardly interact with the surrounding MOF lattice. The influence of water upon the Cu_3_(BTC)_2_ structure was studied in detail by ^1^H and ^13^C MAS NMR spectroscopy [211]. It turned out that this MOF decomposes rather quickly depending on the amount of adsorbed water.

MIL-53—which has already been discussed in the context of ^129^Xe NMR spectroscopy—also changes its structure during water adsorption. The structural changes present in the MIL-53 lattice could be directly probed by MAS NMR spectroscopy [189]. Another flexible MOF, the aforementioned gate-pressure MOF DUT-8(Ni) does not only change its crystal structure, but also its electronic structure during switching between the open and closed (or large pore and narrow pore) form [23,183]. This effect manifests itself in different colors of the two forms. The magnetization is also changed during pore opening/closing. The underlying electronic changes clearly manifest themselves in huge variations of the ^13^C{^1^H} CP MAS NMR signals, especially in the line widths and the chemical shifts as discussed above (see Figure 6). Adsorption-induced differences in NMR spectra are frequently used to obtain information about changes of the MOF lattice, for example about structural differences between the solvated and activated state [31,42,107,117,189,207,212]. Some MOF compounds, especially those containing amino acid linkers, can be regarded as simple model systems for proteins. Rabone *et al.* characterized a peptide-based, flexible MOF by means of XRD methods, MAS NMR spectroscopy, and molecular dynamics simulations [205].

Petersen *et al.* [213] investigated the uptake of ammonia vapour by the MOF Cu_3_(BTC)_2_ using ^1^H and ^13^C MAS NMR spectroscopy. It could be shown that the extensive ammonia sorption on this compound is accompanied by severe loss of porosity and structural decomposition, especially if water is present.

It should also be noted that the adsorption of methanol and acetonitrile resulted in significant structural changes of Cu-MOF (Cu(bpy)(H_2_O)_2_(BF_4_)_2_(bpy); bpy = 4,4'-bipyridine) which could be visualized by ^11^B MAS NMR spectroscopy in combination with EPR [31].

### 3.3. Practical Aspects

Finally, some practical aspects related to the special properties of MOFs should be briefly discussed.

The complete removal of solvent molecules from the samples deserves special attention in order to ensure defined conditions for NMR experiments on activated samples or on loaded samples, *i.e.*, if host–guest interactions with other adsorbed species are investigated. Moreover, the structure of MOFs with flexible lattices can significantly be influenced by residual amounts of solvent molecules. Solvent extraction by processing in supercritical or liquid CO_2_ (“supercritical drying”) turned out to be very efficient in comparison with other established techniques such as thermal evacuation or solvent exchange [191].

**Figure 15:**
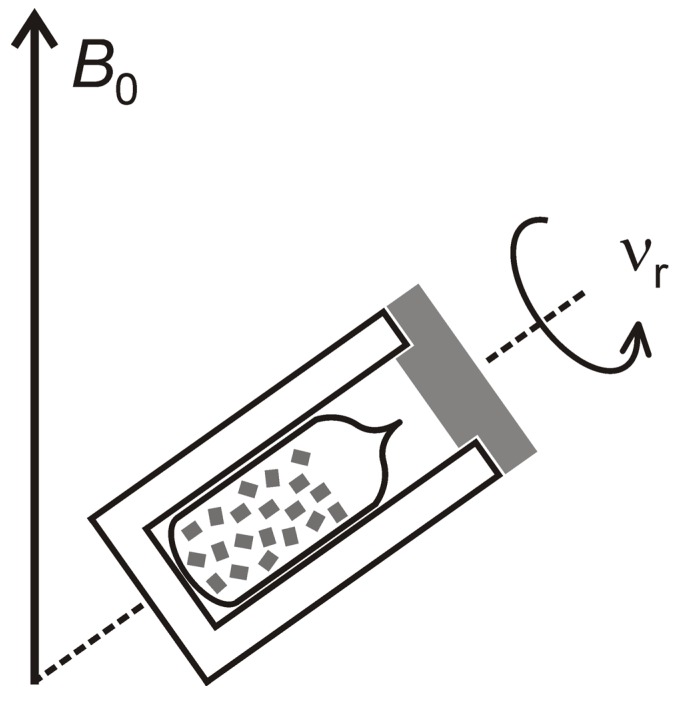
Schematic: MAS NMR of a sample sealed into a glass ampoule.

Much attention is needed for the preparation of the NMR samples because MOFs may be very sensitive to air and/or moisture exposure. For example, the spectrum of UMCM-1 significantly changes even after very short (*ca.* 30 s) air exposure. It is, therefore, necessary to handle such samples at least under an inert atmosphere, e.g., in a glove box. Ideally, the samples can be sealed in glass ampoules even for MAS NMR experiments ([214,215], see below, Figure 15).

**Figure 16:**
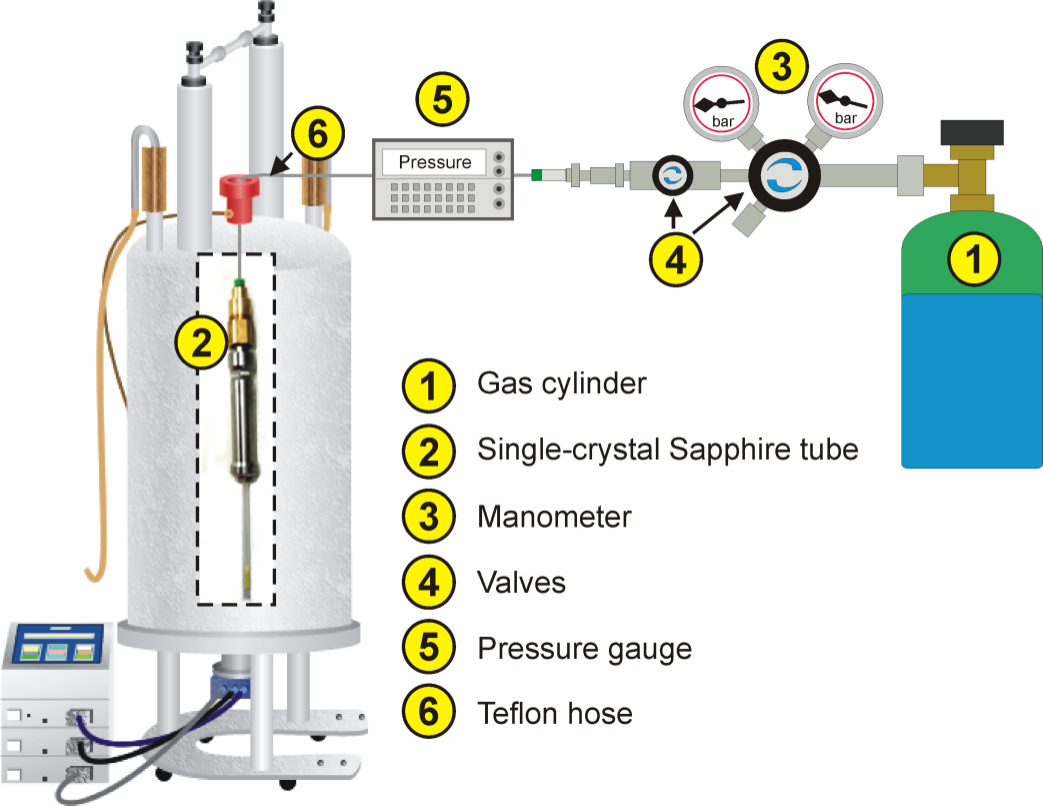
High-pressure apparatus for *in situ* NMR studies of gas adsorption. Reprinted with permission from [183]. Copyright 2011 American Chemical Society.

Solid-state NMR spectroscopic studies of host–guest interactions in porous materials usually require the use of sealed samples in order to ensure that the measurements are carried out under defined conditions. This is particularly necessary if the adsorbed species are gases which desorb readily or if molecules from the atmosphere (especially water molecules) are taken up by the material. Such species may compete with the adsorbed species or may even react with the MOF thus influencing the results of the measurements. If the samples need to be kept in sealed containers during the NMR measurements, two different cases must be considered, namely: (i) without MAS (Figure 16) and (ii) with MAS (Figure 15).

Without MAS, the samples may either be sealed in glass ampoules—provided the pressure is relatively low—or in special high-pressure tubes. Recently, an apparatus for *in situ* high-pressure NMR spectroscopy has been introduced which allows applying defined pressures of gases to solvent-free, *i.e.*, dried, samples inside the magnet under controlled temperatures (see Figure 16, [183]).

With MAS, the samples have to be loaded with defined amounts of the desired gas and sealed in glass ampoules. Note that the ampoules must exhibit a sufficiently good cylindrical symmetry in order to allow sample spinning as is schematically shown in Figure 15 ([214,215]).

## References

[1] Pfeifer H. (1994). NMR of solid surfaces. NMR Basic Princ. Prog..

[2] Brunner E. (1995). Solid state NMR—A powerful tool for the investigation of surface hydroxyl groups in zeolites and their interactions with adsorbed probe molecules. J. Mol. Struct..

[3] Ebener M., Francke V., Günther H. (1997). Solid state ^13^C MAS NMR as a tool for the study of reactions between compounds adsorbed on porous materials. Fresen. J. Anal. Chem..

[4] Watson A.T., Chang C.T.P. (1997). Characterizing porous media with NMR methods. Prog. Nucl. Magn. Reson. Spectrosc..

[5] Capuani S., Alesiani M., Branca R.T., Maraviglia B. (2004). New openings for porous systems research from intermolecular double-quantum NMR. Solid State Nucl. Magn. Reson..

[6] Hunger M., Brunner E. (2004). NMR spectroscopy. Mol. Sieves.

[7] Ashbrook S.E., Smith M.E. (2006). Solid-state ^17^O NMR—An introduction to the background principles and applications to inorganic materials. Chem. Soc. Rev..

[8] Buntkowsky G., Breitzke H., Adamczyk A., Roelofs F., Emmler T., Gedat E., Grünberg B., Xu Y., Limbach H.-H., Shenderovich I., Vyalikh A., Findenegg G. (2007). Structural and dynamical properties of guest molecules confined in mesoporous silica materials revealed by NMR. Phys. Chem. Chem. Phys..

[9] Bonhomme C., Coelho C., Baccile N., Gervais C., Azaïs T., Babonneau F. (2007). Advanced solid state NMR techniques for the characterization of sol-gel-derived materials. Acc. Chem. Res..

[10] Bakhmutov V.I. (2011). Strategies for solid-state NMR studies of materials: From diamagnetic to paramagnetic porous solids. Chem. Rev..

[11] Koller H., Weiss M. (2012). Solid state NMR of porous materials: Zeolites and related materials. Top. Curr. Chem..

[12] Chmelik C., Kärger J. (2010). *In situ* study on molecular diffusion phenomena in nanoporous catalytic solids. Chem. Soc. Rev..

[13] Huang J., Jiang Y., Marthala V.R., Hunger M. (2008). Insight into the mechanisms of the ethylbenzene disproportionation: Transition state shape selectivity on zeolites. J. Am. Chem. Soc..

[14] Ivanova I.I., Kolyagin Y.G. (2010). Impact of *in situ* MAS NMR techniques to the understanding of the mechanisms of zeolite catalyzed reactions. Chem. Soc. Rev..

[15] Zhang W., Xu S., Han X., Bao X. (2012). *In situ* solid-state NMR for heterogeneous catalysis: A joint experimental and theoretical approach. Chem. Soc. Rev..

[16] Blasco T. (2010). Insights into reaction mechanisms in heterogeneous catalysis revealed by *in situ* NMR spectroscopy. Chem. Soc. Rev..

[17] Eddaoudi M., Kim J., Rosi N., Vodak D., Wachter J., O’Keefe M., Yaghi O. (2002). Systematic design of pore size and functionality in isoreticular MOFs and their application in methane storage. Science.

[18] Férey G. (2007). Metal-organic frameworks: The young child of the porous solids family. Stud. Surf. Sci. Catal..

[19] Kaskel S., Schüth F., Sing K.S.W., Weitkamp J. (2002). Porous metal–organic frameworks. Hanbook of Porous Solids.

[20] Kitagawa S., Kitaura R., Noro S.-I. (2004). Functional porous coordination polymers. Angew. Chem. Int. Ed..

[21] Férey G. (2008). Hybrid porous solids: Past, present, future. Chem. Soc. Rev..

[22] Serre C., Millange F., Thouvenot C., Noguès M., Marsolier G., Louër D., Férey G. (2002). Very large breathing effect in the first nanoporous Chromium(III)-based solids: MIL-53 or Cr^III^(OH)·{O_2_C-C_6_H_4_-CO_2_}·{HO_2_C-C_6_H_4_-CO_2_H}*_x_*·H_2_O*_y_*. J. Am. Chem. Soc..

[23] Klein N., Herzog C., Sabo M., Senkovska I., Getzschmann J., Paasch S., Lohe M.R., Brunner E., Kaskel S. (2010). Monitoring adsorption-induced switching by ^129^Xe NMR spectroscopy in a new metal–organic framework Ni_2_(2,6-ndc)_2_(dabco). Phys. Chem. Chem. Phys..

[24] Klein N., Hoffmann H.C., Cadiau A., Getzschmann J., Lohe M.R., Paasch S., Heydenreich T., Adil K., Senkovska I., Brunner E., Kaskel S. (2012). Structural flexibility and intrinsic dynamics in M_2_(2,6-ndc)_2_(dabco) (M = Ni, Co, Cu, Zn) metal–organic frameworks. J. Mater. Chem..

[25] Henke S., Schneemann A., Wütscher A., Fischer R.A. (2012). Directing the breathing behavior of pillared-layered metal–organic frameworks via a systematic library of functionalized linkers bearing flexible substituents. J. Am. Chem. Soc..

[26] Li D., Kaneko K. (2001). Hydrogen bond-regulated microporous nature of copper complex-assembled microcrystals. Chem. Phys. Lett..

[27] Evans O.R., Lin W. (2002). Crystal engineering of NLO materials based on metal–organic coordination networks. Acc. Chem. Res..

[28] Srikanth H., Hajndl R., Moulton B., Zaworotko M. (2003). Magnetic studies of crystal-engineered molecular nanostructures (invited). J. Appl. Phys..

[29] Maspoch D., Ruiz-Molina D., Veciana J. (2004). Magnetic nanoporous coordination polymers. J. Mater. Chem..

[30] Halder G.J., Kepert C.J., Moubaraki B., Murray K.S., Cashion J.D. (2002). Guest-dependent spin crossover in a nanoporous molecular framework material. Science.

[31] Jiang Y., Huang J., Kasumaj B., Jeschke G., Hunger M., Mallat T., Baiker A. (2009). Adsorption-desorption induced structural changes of Cu-MOF evidenced by solid state NMR and EPR spectroscopy. J. Am. Chem. Soc..

[32] Stallmach F., Gröger S., Künzel V., Kärger J., Yaghi O.M., Hesse M., Müller U. (2006). NMR studies on the diffusion of hydrocarbons on the metal–organic framework material MOF-5. Angew. Chem. Int. Ed..

[33] Harris R.K., Wasylishen R.E., Duer M. (2009). NMR Crystallography.

[34] Harris R.K. (2004). NMR crystallography: The use of chemical shifts. Solid State Sci..

[35] Pickard C.J., Mauri F. (2001). All-electron magnetic response with pseudopotentials: NMR chemical shifts. Phys. Rev. B.

[36] Martineau C., Cadiau A., Bouchevreau B., Senker J., Taulelle F., Adil K. (2012). SMARTER crystallography of the fluorinated inorganic–organic compound Zn_3_Al_2_F_12_∙[HAmTAZ]_6_. Dalton Trans..

[37] Gerardy-Montouillout V.V., Malveau C., Tekely P., Olender Z., Luz Z. (1996). ODESSA, a new 1D NMR exchange experiment for chemically equivalent nuclei in rotating solids. J. Magn. Reson. A.

[38] Reichert D., Zimmermann H., Tekely P., Poupko R., Luz Z. (1997). Time-reverse ODESSA. A 1D exchange experiment for rotating solids with several groups of equivalent nuclei. J. Magn. Reson..

[39] Krushelnitsky A., de Azevedo E., Linser R., Reif B., Saalwächter K., Reichert D. (2009). Direct observation of millisecond to second motions in proteins by dipolar CODEX NMR spectroscopy. J. Am. Chem. Soc..

[40] Li W., McDermott A. (2009). Characterization of slow conformational dynamics in solids: Dipolar CODEX. J. Biomol. NMR.

[41] Loiseau T., Muguerra H., Férey G., Haouas M., Taulelle F. (2005). Synthesis and structural characterization of a new open-framework zinc terephthalate Zn_3_(OH)_2_(bdc)_2_·2DEF, with infinite Zn–(µ_3_-OH)–Zn chains. J. Solid State Chem..

[42] Jiang Y., Huang J., Marx S., Kleist W., Hunger M., Baiker A. (2010). Effect of dehydration on the local structure of framework Aluminum atoms in mixed linker MIL-53(Al) materials studied by solid-state NMR spectroscopy. J. Phys. Chem. Lett..

[43] Lieder C., Opelt S., Dyballa M., Henning H., Klemm E., Hunger M. (2010). Adsorbate effect on AlO_4_(OH)_2_ centers in the metal–organic framework MIL-53 investigated by solid-state NMR spectroscopy. J. Phys. Chem. C.

[44] Volkringer C., Loiseau T., Férey G., Morais C.M., Taulelle F., Montouillout V., Massiot D. (2007). Synthesis, crystal structure and ^71^Ga solid state NMR of a MOF-type gallium trimesate (MIL-96) with µ_3_-oxo bridged trinuclear units and a hexagonal 18-ring network. Microporous Mesoporous Mater..

[45] Hajjar R., Volkringer C., Loiseau T., Guillou N., Marrot J., Férey G., Margiolaki I., Fink G., Morais C., Taulelle F. (2011). ^71^Ga slow-CTMAS NMR and crystal structures of MOF-type gallium carboxylates with infinite edge-sharing octahedra chains (MIL-120 and MIL-124). Chem. Mater..

[46] Mowat J.P.S., Miller S.R., Slawin A.M.Z., Seymour V.R., Ashbrook S.E., Wright P.A. (2011). Synthesis, characterization and adsorption properties of microporous scandium carboxylates with rigid and flexible frameworks. Microporous Mesoporous Mater..

[47] Sutrisno A., Terskikh V.V., Shi Q., Song Z., Dong J., Ding S.Y., Wang W., Provost B.R., Daff T.D., Woo T.K., Huang Y. (2012). Characterization of Zn-containing metal–organic frameworks by solid-state ^67^Zn NMR spectroscopy and computational modeling. Chem. Eur. J..

[48] Haouas M., Martineau C., Taulelle F., Wasylishen R.E., Ashbrook S.E., Wimperis S. (2012). Quadrupolar NMR of nanoporous materials. NMR of Quadrupolar Nuclei in Solid Materials.

[49] Fraissard J., Ito T. (1988). ^129^Xe NMR study of adsorbed xenon: A new method for studying zeolites and metal-zeolites. Zeolites.

[50] Andrew E.R., Bradbury A., Eades R.G. (1958). Nuclear magnetic resonance spectra from a crystal rotated at high speed. Nature.

[51] Lowe I.J. (1959). Free induction decays of rotating solids. Phys. Rev. Lett..

[52] Pines A., Gibby M.G., Waugh J.S. (1972). Proton-enhanced nuclear induction spectroscopy: A method for high resolution NMR of dilute spins in solids. J. Chem. Phys..

[53] Pines A., Gibby M.G., Waugh J.S. (1973). Proton-enhanced NMR of dilute spins in solids. J. Chem. Phys..

[54] Metz G., Wu X., Smith S.O. (1994). Ramped-amplitude cross polarization in magic-angle-spinning NMR. J. Magn. Reson. A.

[55] Hediger S., Meier B.H., Kurur N.D., Bodenhausen G., Ernst R.R. (1994). NMR cross polarization by adiabatic passage through the Hartmann-Hahn condition (APHH). Chem. Phys. Lett..

[56] Wu X., Zilm K.W. (1993). Complete spectral editing in CPMAS NMR. J. Magn. Reson..

[57] Lesage A., Steuernagel S., Emsley L. (1998). Carbon-13 spectral editing in Solid-State NMR using heteronuclear scalar couplings. J. Am. Chem. Soc..

[58] Caravatti P., Braunschweiler L., Ernst R.R. (1983). Heteronuclear correlation spectroscopy in rotating solids. Chem. Phys. Lett..

[59] Lesage A., Sakellariou D., Steuernagel S., Emsley L. (1998). Carbon-proton chemical shift correlation in solid-state NMR by through-bond multiple-quantum spectroscopy. J. Am. Chem. Soc..

[60] Lesage A., Emsley L. (2001). Through-bond heteronuclear single-quantum correlation spectroscopy in solid-state NMR, and comparison to other through-bond and through-space experiments. J. Magn. Reson..

[61] Elena B., Lesage A., Steuernagel S., Böckmann A., Emsley L. (2005). Proton to carbon-13 INEPT in solid-state NMR spectroscopy. J. Am. Chem. Soc..

[62] Lesage A., Bardet M., Emsley L. (1999). Through-bond carbon-carbon connectivities in disordered solids by NMR. J. Am. Chem. Soc..

[63] Volkringer C., Loiseau T., Guillou N., Férey G., Haouas M., Taulelle F., Elkaim E., Stock N. (2010). High-throughput aided synthesis of the porous metal–organic framework-type Aluminum pyromellitate, MIL-121, with extra carboxylic acid functionalization. Inorg. Chem..

[64] Cadiau A., Martineau C., Leblanc M., Maisonneuve V., Hémon-Ribaud A., Taulelle F., Adil K. (2011). ZnAlF_5_·[TAZ]: An Al fluorinated MOF of MIL-53(Al) topology with cationic {Zn(1,2,4 triazole)}^2+^ linkers. J. Mater. Chem..

[65] Loiseau T., Lecroq L., Volkringer C., Marrot J., Férey G., Haouas M., Taulelle F., Bourrelly S., Llewellyn P.L., Latroche M. (2006). MIL-96, a porous Aluminum trimesate 3D structure constructed from a hexagonal network of 18-membered rings and *μ*_3_-Oxo-centered trinuclear units. J. Am. Chem. Soc..

[66] Volkringer C., Popov D., Loiseau T., Guillou N., Férey G., Haouas M., Taulelle F., Mellot-Draznieks C., Burghammer M., Riekel C. (2007). A microdiffraction set-up for nanoporous metal–organic-framework-type solids. Nat. Mater..

[67] Volkringer C., Loiseau T., Haouas M., Taulelle F., Popov D., Burghammer M., Riekel C., Zlotea C., Cuevas F., Latroche M., Phanon D., Knöfelv C., Llewellyn P.L., Férey G. (2009). Occurrence of uncommon infinite chains consisting of edge-sharing octahedra in a porous metal organic framework-type Aluminum pyromellitate Al_4_(OH)_8_[C_10_O_8_H_2_] (MIL-120): Synthesis, structure, and gas sorption properties. Chem. Mater..

[68] Devic T., Wagner V., Guillou N., Vimont A., Haouas M., Pascolini M., Serre C., Marrot J., Daturi M., Taulelle F., Férey G. (2011). Synthesis and characterization of a series of porous lanthanide tricarboxylates. Microporous Mesoporous Mater..

[69] Biemmi E., Bein T., Stock N. (2006). Synthesis and characterization of a new metal organic framework structure with a 2D porous system: (H_2_NEt_2_)_2_[Zn_3_(BDC)_4_]·3DEF. Solid State Sci..

[70] Frydman L. (2001). Spin-1/2 and beyond: A perspective in solid state NMR spectroscopy. Ann. Rev. Phys. Chem..

[71] Jerschow A. (2005). From nuclear structure to the quadrupolar NMR interaction and high-resolution spectroscopy. Prog. Nucl. Magn. Reson. Spectrosc..

[72] Bai S., Wang W., Dybowski C. (2010). Solid state NMR spectroscopy. Anal. Chem..

[73] Mehring M., Weberruss V.A. (2001). Object-Oriented Magnetic Resonance: Classes and Objects, Calculations And Computations.

[74] Bennett A.E., Rienstra C.M., Auger M., Lakshmi K.V., Griffin R.G. (1995). Heteronuclear decoupling in rotating solids. J. Chem. Phys..

[75] Fung B.M., Khitrin A.K., Ermolaev K. (2000). An improved broadband decoupling sequence for liquid crystals and solids. J. Magn. Reson..

[76] Fernandez C., Pruski M. (2012). Probing quadrupolar nuclei by solid-state NMR spectroscopy: Recent advances. Top. Curr. Chem..

[77] Llor A., Virlet J. (1988). Towards high-resolution NMR of more nuclei in solids: Sample spinning with time-despendent spinner axis angle. Chem. Phys. Lett..

[78] Chmelka B.F., Mueller K.T., Pines A., Stebbins J., Wu Y., Zwanziger J.W. (1989). Oxygen-17 NMR in solids by dynamic-angle spinning and double rotation. Nature.

[79] Medek A., Harwood J.S., Frydman L. (1995). Multiple-quantum magic-angle spinning NMR: A new method for the study of quadrupolar nuclei in solids. J. Am. Chem. Soc..

[80] Frydman L., Harwood J.S. (1995). Isotropic spectra of half-integer quadrupolar spins from bidimensional magic-angle spinning NMR. J. Am. Chem. Soc..

[81] Goldbourt A., Madhu P.K. (2002). Multiple-quantum magic-angle spinning: High-resolution solid state NMR spectroscopy of half-integer quadrupolar nuclei. Monatsh. Chem..

[82] Gan Z. (2000). Isotropic NMR spectra of half-integer quadrupolar nuclei using satellite transitions and magic-angle spinning. J. Am. Chem. Soc..

[83] Vosegaard T., Massiot D. (2007). High-resolution two-dimensional NMR spectra of half-integer-spin quadrupolar nuclei from one-dimensional projections. Chem. Phys. Lett..

[84] Chabanas M., Quadrelli E.A., Fenet B., Copéret C., Thivolle-Cazat J., Basset J.-M., Lesage A., Emsley L. (2001). Molecular insight into surface organometallic chemistry through the combined use of 2D HETCOR solid-state NMR spectroscopy and silsesquioxane analogues. Angew. Chem. Int. Ed..

[85] Azais T., Hartmeyer G., Quignard S., Laurent G., Babonneau F. (2010). Solution state NMR techniques applied to solid state samples: Characterization of Benzoic acid confined in MCM-41. J. Phys. Chem. C.

[86] Brunner E., Freude D., Gerstein B.C., Pfeifer H. (1990). Residual linewidths of NMR spectra of spin-1/2 systems under magic-angle spinning. J. Magn. Reson..

[87] Brunner E. (1990). Limitations of resolution in the ^1^H magic-angle-spinning nuclear magnetic resonance spectroscopy of zeolites. J. Chem. Soc. Faraday Trans..

[88] Samoson A., Tuherm T., Past J., Reinhold A., Anupõld T., Heinmaa I. (2004). New horizons for magic angle spinning NMR. Top. Curr. Chem..

[89] Bielecki A., Kolbert A.C., Levitt M.H. (1989). Frequency-switched pulse sequences: Homonuclear decoupling and dilute spin NMR in solids. Chem. Phys. Lett..

[90] Vinogradov E., Madhu P.K., Vega S. (1999). High-resolution proton solid-state NMR spectroscopy by phase-modulated Lee-Goldburg experiment. Chem. Phys. Lett..

[91] Lesage A., Sakellariou D., Hediger S., Eléna B., Charmont P., Steuernagel S., Emsley L. (2003). Experimental aspects of proton NMR spectroscopy in solids using phase-modulated homonuclear dipolar decoupling. J. Magn. Reson..

[92] Halse M.E., Emsley L. (2012). A common theory for phase-modulated homonuclear decoupling in solid-state NMR. Phys. Chem. Chem. Phys..

[93] Sakellariou D., Lesage A., Hodgkinson P., Emsley L. (2000). Homonuclear dipolar decoupling in solid-state NMR using continuous phase modulation. Chem. Phys. Lett..

[94] Salager E., Stein R.S., Steuernagel S., Lesage A., Elena B., Emsley L. (2009). Enhanced sensitivity in high-resolution ^1^H solid-state NMR spectroscopy with DUMBO dipolar decoupling under ultra-fast MAS. Chem. Phys. Lett..

[95] Kirillov A.M., Wieczorek S.W., Lis A., Guedes da Silva M.F.C., Florek M., Król J., Staroniewicz Z., Smolénski P., Pombeiro A.J.L. (2011). 1,3,5-Triaza-7-phosphaadamantane-7-oxide (PTA=O): New diamondoid building block for design of three-dimensional metal–organic frameworks. Cryst. Growth Des..

[96] Volkringer C., Meddouri M., Loiseau T., Guillou N., Marrot J., Férey G., Haouas M., Taulelle F., Audebrand N., Latroche M. (2008). The Kagomé Topology of the gallium and indium metal–organic framework types with a MIL-68 structure: Synthesis, XRD, solid-state NMR characterizations, and hydrogen adsorption. Inorg. Chem..

[97] Shi F.N., Cunha-Silva L., Sá Ferreira R.A., Mafra L., Trindade T., Carlos L.D., Almeida Paz F.A., Rocha J. (2008). Interconvertable modular framework and layered Lanthanide(III)-Etidronic acid coordination polymers. J. Am. Chem. Soc..

[98] Malicki N., Mafra L., Quoineaud A.-A., Rocha J., Thibault-Starzyk F., Fernandez C. (2005). Multiplex MQMAS NMR of quadrupolar nuclei. Solid State Nucl. Mag..

[99] Gan Z., Kwak H.-T. (2004). Enhancing MQMAS sensitivity using signals from multiple coherence transfer pathways. J. Magn. Reson..

[100] Bajpe S.R., Kirschhock C.E.A., Aerts A., Breynaert E., Absillis G., Parac-Vogt T.N., Giebeler L., Martens J.A. (2010). Direct observation of molecular-level template action leading to self-assembly of a porous framework. Chem. Eur. J..

[101] Gullion T., Schaefer J. (1989). Rotational-echo double-resonance NMR. J. Magn. Reson..

[102] Levitt M.H., Raleigh D.P., Creuzet F., Griffin R.G. (1990). Theory and simulations of homonuclear spin pairs in rotating solids. J. Chem. Phys..

[103] de Paëpe G. (2012). Dipolar recoupling in magic angle spinning solid-state nuclear magnetic resonance. Ann. Rev. Phys. Chem..

[104] Brouwer D.H., Darton R.J., Morris R.E., Levitt M.H. (2005). A solid-state NMR method for solution of zeolite crystal structures. J. Am. Chem. Soc..

[105] Brouwer D.H., Kristiansen P.E., Fyfe C.A., Levitt M.H. (2005). Symmetry-based ^29^Si dipolar recoupling magic angle spinning NMR spectroscopy: A new method for investigating three-dimensional structures of zeolite frameworks. J. Am. Chem. Soc..

[106] Kristiansen P.E., Mitchell D.J., Evans J.N. (2002). Double-quantum dipolar recoupling at high magic-angle spinning rates. J. Magn. Reson..

[107] Haouas M., Volkringer C., Loiseau T., Férey G., Taulelle F. (2011). Monitoring the activation process of the giant pore MIL-100(Al) by solid state NMR. J. Phys. Chem. C.

[108] Volkringer C., Loiseau T., Guillou N., Férey G., Haouas M., Taulelle F., Audebrand N., Margiolaki I., Popov D., Burghammer M., Riekel C. (2009). Structural transitions and flexibility during dehydration-rehydration process in the MOF-type Aluminum pyromellitate Al_2_(OH)_2_[C_10_O_8_H_2_] (MIL-118). Cryst. Growth Des..

[109] Goesten M.G., Juan-Alcañiz J., Ramos-Fernandez E.V., Gupta K.B.S.S., Stavitski E., van Bekkum H., Gascon J., Kapteijn F. (2011). Sulfation of metal–organic frameworks: Opportunities for acid catalysis and proton conductivity. J. Catal..

[110] Rowsell J.L.C., Yaghi O.M. (2006). Effects of functionalization, catenation, and variation of the metal oxide and organic linking units on the low-pressure hydrogen adsorption properties of metal–organic frameworks. J. Am. Chem. Soc..

[111] Gadzikwa T., Lu G., Stern C.L., Wilson S.R., Hupp J.T., Nguyen S.T. (2008). Covalent surface modification of a metal–organic framework: Selective surface engineering via Cu^I^-catalyzed Huisgen cycloaddition. Chem. Commun..

[112] Tanabe K.K., Wang Z., Cohen S.M. (2008). Systematic functionalization of a metal–organic framework via a postsynthetic modification approach. J. Am. Chem. Soc..

[113] Burrows A.D., Frost C.G., Mahon M.F., Richardson C. (2009). Sulfur-tagged metal–organic frameworks and their post-synthetic oxidation. Chem. Commun..

[114] Mavrandonakis A., Klontzas E., Tylianakis E., Froudakis G.E. (2009). Enhancement of hydrogen adsorption in metal–organic frameworks by the incorporation of the sulfonate group and Li cations: A multiscale computational study. J. Am. Chem. Soc..

[115] Ingleson M.J., Heck R., Gould J.A., Rosseinsky M.J. (2009). Nitric oxide chemisorption in a postsynthetically modified metal–organic framework. Inorg. Chem..

[116] Henke S., Schmid R., Grunwaldt J.-D., Fischer R.A. (2010). Flexibility and sorption selectivity in rigid metal–organic frameworks: The impact of ether-functionalised linkers. Chem. Eur. J..

[117] Savonnet M., Bazer-Bachi D., Bats N., Perez-Pellitero J., Jeanneau E., Lecocq V., Pinel C., Farrusseng D. (2010). Generic postfunctionalization route from amino-derived metal–organic frameworks. J. Am. Chem. Soc..

[118] Britt D., Lee C., Uribe-Romo F.J., Furukawa H., Yaghi O.M. (2010). Ring-opening reactions within metal–organic frameworks. Inorg. Chem..

[119] Yang Q., Wiersum A.D., Llewellyn P.L., Guillerm V., Serre C., Maurin G. (2011). Functionalizing porous zirconium terephthalate UiO-66(Zr) for natural gas upgrading: A computational exploration. Chem. Commun..

[120] Tanabe K.K., Cohen S.M. (2011). Postsynthetic modification of metal–organic frameworks—A progress report. Chem. Soc. Rev..

[121] Dröge T., Notzon A., Fröhlich R., Glorius F. (2011). Palladium-catalyzed C-H Bond functionalization of a metal–organic framework (MOF): Mild, selective, and efficient. Chem. Eur. J..

[122] Kim M., Cahill J.F., Su Y., Prather K.A., Cohen S.M. (2012). Postsynthetic ligand exchange as a route to functionalization of “inert” metal–organic frameworks. Chem. Sci..

[123] Halls J.E., Hernán-Gómez A., Burrows A.D., Marken F. (2012). Metal-organic frameworks post-synthetically modified with ferrocenyl groups: Framework effects on redox processes and surface conduction. Dalton Trans..

[124] Savonnet M., Camarata A., Canivet J., Bazer-Bachi D., Bats N., Lecocq V., Pinel C., Farrusseng D. (2012). Tailoring metal–organic framework catalysts by click chemistry. Dalton Trans..

[125] Ahnfeldt T., Gunzelmann D., Wack J., Senker J., Stock N. (2012). Controlled modification of the inorganic and organic bricks in an Al-based MOF by direct and post-synthetic synthesis routes. Cryst. Eng. Commun..

[126] Proch S., Herrmannsdörfer J., Kempe R., Kern C., Jess A., Seyfarth L., Senker J. (2008). Pt@MOF-177: Synthesis, room-temperature hydrogen storage and oxidation catalysis. Chem. Eur. J..

[127] Wang Z., Cohen S.M. (2007). Postsynthetic covalent modification of a neutral metal–organic framework. J. Am. Chem. Soc..

[128] Roy P., Schaate A., Behrens P., Godt A. (2012). Post-Synthetic Modification of Zr-Metal-Organic Frameworks through Cycloaddition Reactions. Chem. Eur. J..

[129] Garibay S.J., Wang Z., Tanabe K.K., Cohen S.M. (2009). Postsynthetic modification: A versatile approach toward multifunctional metal–organic frameworks. Inorg. Chem..

[130] Wang Z., Tanabe K.K., Cohen S.M. (2009). Accessing postsynthetic modification in a series of metal–organic frameworks and the influence of framework topology on reactivity. Inorg. Chem..

[131] Garibay S.J., Wang Z., Cohen S.M. (2010). Evaluation of heterogeneous metal–organic framework organocatalysts prepared by postsynthetic modification. Inorg. Chem..

[132] Mowat J.P.S., Miller S.R., Griffin J.M., Seymour V.R., Ashbrook S.E., Thompson S.P., Fairen-Jimenez D., Banu A.-M., Düren T., Wright P.A. (2011). Structural chemistry, monoclinic-to-orthorhombic phase transition, and CO_2_ adsorption behavior of the small pore scandium terephthalate, Sc_2_(O_2_CC_6_H_4_CO_2_)_3_, and its nitro- and amino-functionalized derivatives. Inorg. Chem..

[133] Ahnfeldt T., Gunzelmann D., Loiseau T., Hirsemann D., Senker J., Férey G., Stock N. (2009). Synthesis and modification of a functionalized 3D open-framework structure with MIL-53 topology. Inorg. Chem..

[134] Bernt S., Feyand M., Modrow A., Wack J., Senker J., Stock N. (2011). [Zn(C_3_H_3_N_2_)(C_3_H_2_N_2_–N=N–C_6_H_5_)], a mixed-linker ZIF containing a photoswitchable phenylazo group. Eur. J. Inorg. Chem..

[135] Morris W., Doonan C.J., Yaghi O.M. (2011). Postsynthetic modification of a metal–organic framework for stabilization of a hemiaminal and ammonia uptake. Inorg. Chem..

[136] Marx S., Kleist W., Huang J., Maciejewski M., Baiker A. (2010). Tuning functional sites and thermal stability of mixed-linker MOFs based on MIL-53(Al). Dalton Trans..

[137] Padmanaban W., Müller P., Lieder C., Gedrich K., Grünker R., Bon V., Senkovska I., Baumgärtner S., Opelt S., Paasch S., Brunner E., Glorius F., Klemm E., Kaskel S. (2011). Application of a chiral metal–organic framework in enantioselective separation. Chem. Commun..

[138] Hoffmann H.C., Paasch S., Müller P., Senkovska I., Padmanaban M., Glorius F., Kaskel S., Brunner E. (2012). Chiral recognition in metal–organic frameworks studied by solid-state NMR spectroscopy using chiral solvating agents. Chem. Commun..

[139] Wenzel T.J. (2007). Discrimination of Chiral Compounds Using NMR Spectroscopy.

[140] Haouas M., Volkringer C., Loiseau T., Férey G., Taulelle F. (2009). The extra-framework sub-lattice of the metal–organic framework MIL-110: A solid-state NMR investigation. Chem. Eur. J..

[141] Ravon U., Savonnet M., Aguado S., Domine M.E., Janneau E., Farrusseng D. (2010). Engineering of coordination polymers for shape selective alkylation of large aromatics and the role of defects. Microporous Mesoporous Mater..

[142] Grajciar L., Bludský O., Nachtigall P. (2010). Water adsorption on coordinatively unsaturated sites in CuBTC MOF. J. Phys. Chem. Lett..

[143] Hong D.-Y., Hwang Y.K., Serre C., Férey G., Chang J.-S. (2009). Porous chromium terephthalate MIL-101 with coordinatively unsaturated sites: Surface functionalization, encapsulation, sorption and catalysis. Adv. Funct. Mater..

[144] Volkringer C., Popov D., Loiseau T., Férey G., Burghammer M., Riekel C., Haouas M., Taulelle F. (2009). Synthesis, single-crystal X-ray microdiffraction, and NMR characterizations of the giant pore metal–organic framework aluminum trimesate MIL-100. Chem. Mater..

[145] Banerjee D., Kim S.J., Li W., Wu H., Li J., Borkowski L.A., Philips B.L., Parise J.B. (2010). Synthesis and structural characterization of a 3-D lithium based metal–organic framework showing dynamic structural behavior. Cryst. Growth Des..

[146] Yang S., Martin G.S.B., Titman J.J., Blake A.J., Allan D.R., Champness N.R., Schröder M. (2011). Pore with gate: Enhancement of the isosteric heat of adsorption of dihydrogen via postsynthetic cation exchange in metal–organic frameworks. Inorg. Chem..

[147] Hurd J.A., Vaidhyanathan R., Thangadurai V., Ratcliffe C.I., Moudrakovski I.L., Shimizu G.K.H. (2009). Anhydrous proton conduction at 150 °C in a crystalline metal–organic framework. Nat. Chem..

[148] Bureekaew S., Horike S., Higuchi M., Mizuno M., Kawamura T., Tanaka D., Yanai N., Kitagawa S. (2009). One-dimensional imidazole aggregate in aluminium porous coordination polymers with high proton conductivity. Nat. Mater..

[149] Taylor J.M., Mah R.K., Moudrakovski I.L., Ratcliffe C.I., Vaidhyanathan R., Shimizu G.K.H. (2010). Facile proton conduction via ordered water molecules in a phosphonate metal–organic framework. J. Am. Chem. Soc..

[150] Gabuda S.P., Kozlova S.G., Samsonenko D.G., Dybtsev D.N., Fedin V.P. (2011). Quantum rotations and chiral polarization of qubit prototype molecules in a highly porous metal–organic framework: ^1^H NMR T_1_ study. J. Phys. Chem. C.

[151] Morris W., Taylor R.E., Dybowski C., Yaghi O.M., Garcia-Garibay M.A. (2011). Framework mobility in the metal–organic framework crystal IRMOF-3: Evidence for aromatic ring and amine rotation. J. Mol. Struct..

[152] Gonzalez J., Devi R.N., Tunstall D.P., Cox P.A., Wright P.A. (2005). Deuterium NMR studies of framework and guest mobility in the metal–organic framework compound MOF-5, Zn_4_O(O_2_CC_6_H_4_CO_2_)_3_. Microporous Mesoporous Mater..

[153] Horike S., Matsuda R., Tanaka D., Matsubara S., Mizuno M., Endo K., Kitagawa S. (2006). Dynamic motion of building blocks in porous coordination polymers. Angew. Chem. Int. Ed..

[154] Gould S.L., Tranchemontagne D., Yaghi O.M., Garcia-Garibay M.A. (2008). Amphidynamic character of crystalline MOF-5: Rotational dynamics of terephthalate phenylenes in a free-volume, sterically unhindered environment. J. Am. Chem. Soc..

[155] Kolokolov D.I., Jobic H., Stepanov A.G., Guillerm V., Devic T., Serre C., Férey G. (2010). Dynamics of benzene rings in MIL-53(Cr) and MIL-47(V) frameworks studied by ^2^H NMR spectroscopy. Angew. Chem. Int. Ed..

[156] Abragam A., Goldman M. (1978). Principles of dynamic nuclear polarization. Rep. Prog. Phys..

[157] Maly T., Debelouchina G.T., Bajaj V.S., Hu K.-N., Joo C.-G., Mak-Jurkauskas M.L., Sirigiri J.R., van der Wel P.C.A., Herzfeld J., Temkin R.J., Griffin R.G. (2008). Dynamic nuclear polarization at high magnetic fields. J. Chem. Phys..

[158] Griesinger C., Bennati M., Vieth H.M., Luchinat C., Parigi G., Höfer P., Engelke F., Glaser S.J., Denysenkov S.J., Prisner T.G. (2012). Dynamic nuclear polarization at high magnetic fields in liquids. Prog. Nucl. Magn. Reson. Spectrosc..

[159] Matsuki Y., Maly T., Ouari O., Karoui H., Le Moigne F., Rizzato E., Lyubenova S., Herzfeld J., Prisner T., Tordo P., Griffin R.G. (2009). Dynamic nuclear polarization with a rigid biradical. Angew. Chem. Int. Ed..

[160] van der Wel P., Hu K.-N., Lewandowski J., Griffin R. (2006). Dynamic nuclear polarization of amyloidogenic peptide nanocrystals: GNNQQNNY, a core segment of the yeast prion prion protein sup35p. J. Am. Chem. Soc..

[161] Rosay M., Tometich L., Pawsey S., Bader R., Schauwecker R., Blank M., Borchard P.M., Cauffman S.R., Felch K.L., Weber R.T., Temkin R.J., Griffin R.G., Maas W.E. (2010). Solid-state dynamic nuclear polarization at 263 GHz: Spectrometer design and experimental results. Phys. Chem. Chem. Phys..

[162] Renault M., Pawsey S., Bos M.P., Koers E.J., Nand D., Tommassen-van Boxtel R., Rosay M., Tommassen J., Maas W.E., Baldus M. (2012). Solid-state NMR spectroscopy on cellular preparations enhanced by dynamic nuclear polarization. Angew. Chem. Int. Ed..

[163] Rossini A.J., Zagdoun A., Lelli M., Canivet J., Aguado S., Ouari O., Tordo P., Rosay M., Maas W.E., Copéret C., Farrusseng D., Emsley L., Lesage A. (2012). Dynamic nuclear polarization enhanced solid-state NMR spectroscopy of functionalized metal–organic frameworks. Angew. Chem..

[164] Ito T., Fraissard J. (1982). ^129^Xe NMR study of xenon adsorbed on Y zeolites. J. Chem. Phys..

[165] Raftery D., Long H., Meersmann T., Grandinetti P.J., Reven L., Pines A. (1991). High-field NMR of adsorbed xenon polarized by laser pumping. Phys. Rev. Lett..

[166] Raftery D., MacNamara E., Fisher G., Rice C.V., Smith J. (1997). Optical pumping and magic angle spinning: sensitivity and resolution enhancement for surface NMR obtained with laser-polarized xenon. J. Am. Chem. Soc..

[167] Brunner E., Seydoux R., Haake M., Pines A., Reimer J. (1998). Surface NMR using laser-polarized ^129^Xe under magic-angle spinning conditions. J. Magn. Reson..

[168] Brunner E. (1999). Enhancement of surface and biological NMR by laser-polarized xenon. Concept. Magn. Res..

[169] Ripmeester J.A., Ratcliffe C.I. (1990). On the application of ^129^Xe NMR to the study of microporous solids. J. Phys. Chem..

[170] Springuel-Huet M.-A., Bonardet J.L., Gédéon A., Fraissard J. (1997). ^129^Xe NMR for studying surface heterogeneity: Well-known facts and new findings. Langmuir.

[171] Ratcliffe C.I. (1998). Xenon NMR. Annu. Rep. NMR Spectrosc..

[172] Moudrakovski I., Soldatov D.V., Ripmeester J.A., Sears D.N., Jameson C.J. (2004). Xe NMR lineshapes in channels of peptide molecular crystals. PNAS.

[173] Raftery D. (2006). Xenon NMR spectroscopy. Ann. Rep. NMR Spectrosc..

[174] Sozzani P., Comotti A., Simonutti R., Meersmann T., Logan J.W., Pines A. (2000). A porous crystalline molecular solid explored by hyperpolarized xenon. Angew. Chem. Int. Ed..

[175] Comotti A., Bracco S., Valsesia P., Ferretti L., Sozzani P. (2007). 2D multinuclear NMR, hyperpolarized xenon and gas storage in organosilica nanochannels with crystalline order in the walls. J. Am. Chem. Soc..

[176] Cheng C.-Y., Bowers C.R. (2007). Direct observation of atoms entering and exiting L-Alanyl-L-valine nanotubes by hyperpolarized ^129^Xe NMR. J. Am. Chem. Soc..

[177] Cheng C.-Y., Stamatatos T.C., Christou G., Bowers C.R. (2010). Molecular wheels as nanoporous materials: Differing modes of gas diffusion through Ga_10_ and Ga_18_ wheels probed by hyperpolarized ^129^Xe NMR spectroscopy. J. Am. Chem. Soc..

[178] Terskikh V.V., Moudrakovski I.L., Breeze S.R., Lang S., Ratcliffe C.I., Ripmeester J.A., Sayari A. (2002). A general correlation for the ^129^Xe NMR chemical shift-pore size relationship in porous silica-based materials. Langmuir.

[179] Böhlmann W., Pöppl A., Sabo M., Kaskel S. (2006). Characterization of the metal–organic framework compound Cu_3_(benzene 1,3,5-tricarboxylate)_2_ by means of ^129^Xe nuclear magnetic and electron paramagnetic resonance spectroscopy. J. Phys. Chem. B.

[180] Ueda T., Kurokawa K., Eguchi T., Kachi-Terajima C., Takamizawa S. (2007). Local structure and xenon adsorption behavior of metal–organic framework system [M_2_(O_2_CPh)_4_(pyz)]_n_ (M = Rh and Cu) as studied with use of single-crystal X-ray diffraction, adsorption isotherm, and xenon-129 NMR. J. Phys. Chem. C.

[181] Ooms K.J., Wasylishen R.E. (2007). ^129^Xe NMR study of xenon in iso-reticular metal–organic frameworks. Microporous Mesoporous Mater..

[182] Springuel-Huet M.-A., Nossov A., Adem Z., Guenneau F., Volkringer C., Loiseau T., Férey G., Gédéon A. (2010). ^129^Xe NMR study of the framework flexibility of the porous hybrid MIL-53(Al). J. Am. Chem. Soc..

[183] Hoffmann H.C., Assfour B., Epperlein F., Klein N., Paasch S., Senkovska I., Kaskel S., Seifert G., Brunner E. (2011). High-pressure *in Situ*
^129^Xe NMR spectroscopy and computer simulations of breathing transitions in the metal–organic framework Ni_2_(2,6-ndc)_2_(dabco) (DUT-8(Ni)). J. Am. Chem. Soc..

[184] Demarquay J., Fraissard J. (1987). ^129^Xe NMR of xenon adsorbed on zeolites: Relationship between the chemical shift and the void space. Chem. Phys. Lett..

[185] Koh K., Wong-Foy A.G., Matzger A.J. (2008). A crystalline mesoporous coordination polymer with high microporosity. Angew. Chem. Int. Ed..

[186] Jameson A.K., Jameson C., Gutowsky H.S. (1970). Density dependence of ^129^Xe chemical shifts in mixtures of xenon and other gases. J. Chem. Phys..

[187] Baumer D., Fink A., Brunner E. (2003). Measurement of the ^129^Xe NMR chemical shift of supercritical xenon. Z. Phys. Chem..

[188] Springuel-Huet M.-A., Fraissard J. (1989). ^129^Xe NMR of xenon on the molecular sieves AlPO_4–1_1 and SAPO-11: Chemical shift anisotropy related to the asymmetry of the adsorption zones. Chem. Phys. Lett..

[189] Loiseau T., Serre C., Huguenard C., Fink G., Taulelle F., Henry M., Bataille T., Férey G. (2004). A rationale for the large breathing of the porous aluminum terephthalate (MIL-53) upon hydration. Chem. Eur. J..

[190] Boutin A., Springuel-Huet M.-A., Nossov A., Gédéon A., Loiseau T., Volkringer C., Férey G., Coudert F.-X., Fuchs A.H. (2009). Breathing transitions in MIL-53(Al) metal–organic framework upon xenon adsorption. Angew. Chem. Int. Ed..

[191] Nelson A.P., Farha O.K., Mulfort K.L., Hupp J.T. (2009). Supercritical processing as a route to high internal surface areas and permanent microporosity in metal–organic framework materials. J. Am. Chem. Soc..

[192] Walker A.M., Civalleri B., Slater B., Mellot-Draznieks C., Corà F., Zicovich-Wilson C.M., Román-Pérez G., Soler J.M., Gale J.D. (2010). Flexibility in a metal–organic framework material controlled by weak dispersion forces: The bistability of MIL-53(Al). Angew. Chem. Int. Ed..

[193] Salles F., Ghoufi A., Maurin G., Bell R.G., Mellot-Draznieks C., Férey G. (2008). Molecular dynamics simulations of breathing MOFs: Structural transformations of MIL-53(Cr) upon thermal activation and CO_2_ adsorption. Angew. Chem. Int. Ed..

[194] Coudert F.-X., Jeffroy M., Fuchs A.H., Boutin A., Mellot-Draznieks C. (2008). Thermodynamics of guest-induced structural transitions in hybrid organic-inorganic frameworks. J. Am. Chem. Soc..

[195] Coombes D.S., Corà F., Mellot-Draznieks C., Bell R.G. (2009). Sorption-induced breathing in the flexible metal organic framework CrMIL-53: Force-field simulations and electronic structure analysis. J. Phys. Chem. C.

[196] Stejskal E.O., Schaefer J., Henis J.M.S., Tripodi M.K. (1974). Magic-angle carbon-13 NMR study of CO_2_ adsorbed on some molecular sieves. J. Chem. Phys..

[197] Omi H., Ueda T., Miyakubo K., Eguchi T. (2005). Dynamics of CO_2_ molecules confined in the micropores of solids as studied by ^13^C NMR. Appl. Surf. Sci..

[198] Pinto M.L., Mafra L., Guil J.M., Pires J., Rocha J. (2011). Adsorption and activation of CO_2_ by amine-modified nanoporous materials studied by solid-state NMR and ^13^CO_2_ adsorption. Chem. Mater..

[199] Beeler A.J., Orendt A.M., Grant D.M., Cutts P.W., Michl J., Zilm K.W., Downing J.W., Facelli J.C., Schindler M.S., Kutzelnigg W. (1984). Low-temperature ^13^C magnetic resonance in solids. 3. Linear and pseudolinear molecules. J. Am. Chem. Soc..

[200] Ripmeester J.A., Ratcliffe C.I. (1998). The Diverse nature of dodecahedral cages in clathrate hydrates as revealed by ^129^Xe- and ^13^C-NMR spectroscopy: CO_2_ as a small-cage guest. Energy Fuels.

[201] Kong X., Scott E., Ding W., Mason J.A., Long J.R., Reimer J.A. (2012). CO_2_ dynamics in a metal–organic framework with open metal sites. J. Am. Chem. Soc..

[202] Massiot D., Fayon F., Capron M., King I., Le Calvé S., Alonso B., Durand J.-O., Bujoli B., Gan Z., Hoatson G. (2002). Modelling one- and two-dimensional solid-state NMR spectra. Magn. Reson. Chem..

[203] Gassensmith J.J., Furukawa H., Smaldone R.A., Forgan R.S., Botros Y.Y., Yaghi O.M., Stoddart J.F. (2011). Strong and Reversible binding of carbon dioxide in a green metal–organic framework. J. Am. Chem. Soc..

[204] Grzech A., Yang J., Dingemans T.J., Srinivasan S., Magusin P.C.M.M., Mulder F.M. (2011). Irreversible high-temperature hydrogen interaction with the metal organic framework Cu_3_(BTC)_2_. J. Phys. Chem. C.

[205] Rabone J., Yue Y.-F., Chong S.Y., Stylianou K.C., Bacsa J., Bradshaw D., Darling G.R., Berry N.G., Khimyak Y.Z., Ganin A.Y., Wiper P., Claridge J.B., Rosseinsky M.J. (2010). An adaptable peptide-based porous material. Science.

[206] García-Ricard O.J., Fu R., Hernández-Maldonado A.J. (2011). Thermally induced changes in a porous coordination polymer {Cu_2_(pyrazine-2,3-dicarboxylate)_2_(4,4’-bipyridine)} studied via *in Situ* X-ray diffraction and ^13^C cross-polarization magic angle spinning nuclear magnetic resonance spectroscopy. J. Phys. Chem. C.

[207] Esken D., Zhang X., Lebedev O.I., Schröder F., Fischer R.A. (2009). Pd@MOF-5: Limitations of gas-phase infiltration and solution impregnation of [Zn_4_O(bdc)_3_] (MOF-5) with metal–organic palladium precursors for loading with Pd nanoparticles. J. Mater. Chem..

[208] Meilikhov M., Yusenko K., Fischer R.A. (2010). Incorporation of metallocenes into the channel structured metal–organic frameworks MIL-53(Al) and MIL-47(V). Dalton Trans..

[209] Su C.-Y., Goforth A.M., Smith M.D., Pellechia P.J., zur Loye H.-C. (2004). exceptionally stable, hollow tubular metal–organic architectures: Synthesis, characterization, and solid-state transformation study. J. Am. Chem. Soc..

[210] Besara T., Jain P., Dalal N.S., Kuhns P.L., Reyes A.P., Kroto H.W., Cheetham A.K. (2011). Mechanism of the order*-*disorder phase transition, and glassy behavior in the metal–organic framework [(CH_3_)_2_NH_2_]Zn(HCOO)_3_. PNAS.

[211] Gul-E-Noor F., Jee B., Pöppl A., Hartmann M., Himsl D., Bertmer M. (2011). Effects of varying water adsorption on a Cu_3_(BTC)_2_ metal–organic framework (MOF) as studied by ^1^H and ^13^C solid-state NMR spectroscopy. Phys. Chem. Chem. Phys..

[212] Gabuda S.P., Kozlova S.G., Drebuschak V.A., Dybtsev D.N., Fedin V.P. (2008). Dynamic Pseudo Jahn-Teller effect and the phase transition induced by absorption of molecules in metal–organic nanotube framework. J. Phys. Chem. C.

[213] Petersen G.W., Wagner G.W., Balboa A., Mahle J., Sewell T., Karwacki C.J. (2009). Ammonia vapor removal by Cu_3_(BTC)_2_ and its characterization by MAS NMR. J. Phys. Chem. C.

[214] Freude D., Hunger M., Pfeifer H. (1982). Study of Brønsted acidity of zeolites using high-resolution proton magnetic resonance with magic-angle spinning. Chem. Phys. Lett..

[215] Pfeifer H., Freude D., Hunger M. (1985). Nuclear magnetic resonance studies on the acidity of zeolites and related catalysts. Zeolites.

